# Causality of particulate matter on cardiovascular diseases and cardiovascular biomarkers

**DOI:** 10.3389/fpubh.2023.1201479

**Published:** 2023-09-01

**Authors:** Qiubo Wang, Zhimiao Wang, Mingyou Chen, Wei Mu, Zhenxing Xu, Mei Xue

**Affiliations:** ^1^Department of Cardiology, The First Affiliated Hospital of Shandong First Medical University and Shandong Provincial Qianfoshan Hospital, Shandong Medicine and Health Key Laboratory of Cardiac Electrophysiology and Arrhythmia, Jinan, China; ^2^Shandong First Medical University and Shandong Academy of Medical Sciences, Jinan, China

**Keywords:** particulate matter, cardiovascular diseases, cardiovascular biomarkers, Mendelian randomization, causal association

## Abstract

**Background:**

Previous observational studies have shown that the prevalence of cardiovascular diseases (CVDs) is related to particulate matter (PM). However, given the methodological limitations of conventional observational research, it is difficult to identify causality conclusively. To explore the causality of PM on CVDs and cardiovascular biomarkers, we conducted a Mendelian randomization (MR) analysis.

**Method:**

In this study, we obtained summary-level data for CVDs and cardiovascular biomarkers including atrial fibrillation (AF), heart failure (HF), myocardial infarction (MI), ischemic stroke (IS), stroke subtypes, body mass index (BMI), lipid traits, fasting glucose, fasting insulin, and blood pressure from several large genome-wide association studies (GWASs). Then we used two-sample MR to assess the causality of PM on CVDs and cardiovascular biomarkers, 16 single nucleotide polymorphisms (SNPs) for PM2.5 and 6 SNPs for PM10 were obtained from UK Biobank participants. Inverse variance weighting (IVW) analyses under the fixed effects model were used as the main analytical method to calculate MR Estimates, followed by multiple sensitivity analyses to confirm the robustness of the results.

**Results:**

Our study revealed increases in PM2.5 concentration were significantly related to a higher risk of MI (odds ratio (OR), 2.578; 95% confidence interval (CI), 1.611–4.127; *p* = 7.920 × 10^−5^). Suggestive evidence was found between PM10 concentration and HF (OR, 2.015; 95% CI, 1.082–3.753; *p* = 0.027) and IS (OR, 2.279; 95% CI,1.099–4.723; p = 0.027). There was no evidence for an effect of PM concentration on other CVDs. Furthermore, PM2.5 concentration increases were significantly associated with increases in triglyceride (TG) (OR, 1.426; 95% CI, 1.133–1.795; *p* = 2.469 × 10^−3^) and decreases in high-density lipoprotein cholesterol (HDL-C) (OR, 0.779; 95% CI, 0.615–0.986; *p* = 0.038). The PM10 concentration increases were also closely related to the decreases in HDL-C (OR, 0.563; 95% CI, 0.366–0.865; *p* = 8.756 × 10^−3^). We observed no causal effect of PM on other cardiovascular biomarkers.

**Conclusion:**

At the genetic level, our study suggested the causality of PM2.5 on MI, TG, as well HDL-C, and revealed the causality of PM10 on HF, IS, and HDL-C. Our findings indicated the need for continued improvements in air pollution abatement for CVDs prevention.

## 1. Introduction

Globally, one in three deaths is caused by cardiovascular diseases (CVDs) which place a heavy burden on health systems (1). It is essential to find ways to prevent and treat CVDs to reduce their global burden. It’s been a hot topic lately the connection between air pollution and CVDs. The increasing urbanization of the world has led to the exposure of more than 90% of the world’s population to levels of air pollution that exceed the guidelines set by the World Health Organization. As a result of air pollution, both developed and developing countries are facing serious public health concerns. Exposure to outside air pollution is generally identified as a challenge for public health agencies and physicians, especially to fine particulate matter (PM). PM are made up of an intricate mixture of liquid and solid particles, as well as inorganic and organic compounds. Particles are generally classified by size into coarse (aerodynamic diameter < 10 μm; PM10), fine (diameter < 2.5 μm; PM2.5) fractions (2). Due to its high level of danger for human health, PM can be used as a reliable proxy for ambient air pollution related morbidity and mortality (3). During the past decade, researchers have examined the association between PM and human health using epidemiological methods (4–6). Furthermore, despite the fact that PM exposure used to be thought to pose a threat to health, especially the lungs (7), the comprehensive clue now shows the greatest inverse impact occurs in the cardiovascular system (8, 9). A cross-sectional study undertaken in Ahvaz megacity showed that exposure to air pollution is significantly associated with cardiovascular mortality (CM), hospital admissions for cardiovascular disease (HACD), and hospital admission for respiratory disease (HARD) (10). Besides, a study by Dastoorpoor et al. also showed that a higher concentration of PM10 is associated with an increased risk of hospitalization for CVDs (11). Similarly, Moradi and his colleagues noted that exposure to PM, even at low concentrations, is related to an increased risk of cardiovascular diseases hospitalizations (12). Furthermore, several studies also demonstrated that PM increases the risk of CVDs such as atrial fibrillation (AF), heart failure (HF), myocardial infarction (MI), and ischemic stroke (IS) (13–16). However, several studies reported inconclusive conclusions (17–21). In addition, most of the evidence is based on observational studies. Due to residual confounding and reverse causation, these studies have difficulty identifying causality conclusively.

When evaluating the health risks of air pollutant exposure, individual health risks may be better comprehended by considering genetic diversity. It is becoming clearer that the impacts of air pollution vary by individual, with some populations being more vulnerable to its adverse effects. Genetic predisposition appears to play a crucial role in response to air pollution (22). Previous studies have revealed that exposure to PM2.5 causes negative health consequences driven by alteration of gene expression (23, 24). A study conducted by Yao et al. showed that participants with 2 alleles of SIRT1_391 can counteract the adverse effect of PM2.5 and reduce the 26.1% risk of premature mortality (25). Poursafa et al. revealed that synergistic effect of the TM-33G / A polymorphism and air pollutants on factors associated with the onset of atherosclerosis (26). To analyze the effect of genetic polymorphisms and PM on CVDs and cardiovascular biomarkers, we conduct Mendelian randomization (MR) analysis to examine the relationship between them by using data from the publicly available genome-wide association study (GWAS) and UK Biobank.

## 2. Method

### 2.1. Study design

**Figure 1:**
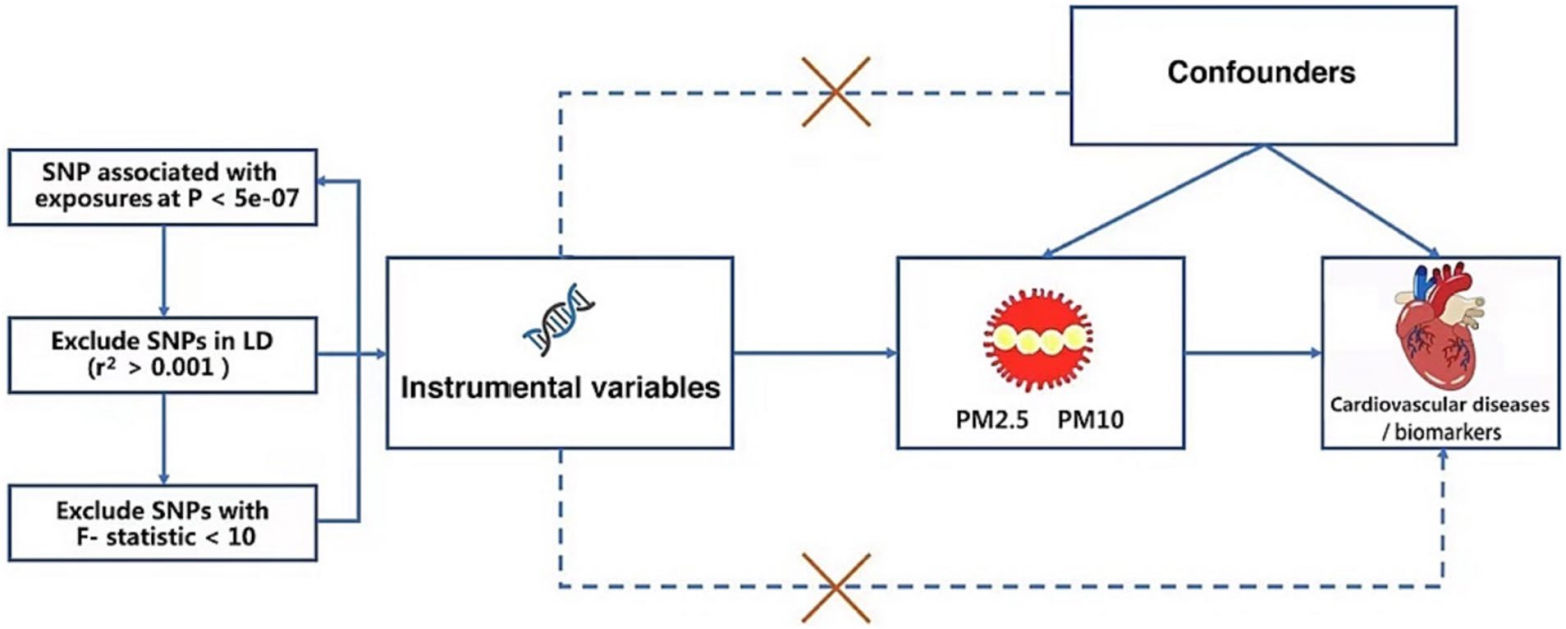
The specific steps for selecting SNPs and three crucial hypotheses of the Mendelian randomization study. PM, particulate matter. SNP, single nucleotide polymorphism.

MR analysis is applied to investigate the relationship between exposure and outcome, which can provide robust causality by utilizing one or multiple genetic variants, such as single nucleotide polymorphisms (SNPs) (27, 28). The MR study was built on the Mendelian inheritance rule, which states that the parents’ genetic alleles are randomly dispersed to the descendants during the process of meiosis, which is supposed to be equivalent to RCT. As a result, the MR design can be used to account for some intractable questions of causality due to high costs or ethical issues, avoiding the biases of observational studies. Therefore, this study intends to examine the causal relationships of PM with CVDs and cardiovascular biomarkers by MR analysis. CVDs included AF, HF, MI, IS, and stroke subtypes. Cardiovascular biomarkers contained body mass index (BMI), triglyceride (TG), high-density lipoprotein cholesterol (HDL-C), low-density lipoprotein cholesterol (LDL-C), fasting glucose, fasting insulin, diastolic blood pressure (DBP), and systolic blood pressure (SBP). MR analysis must satisfy three assumptions. First, IVs must be closely related to exposure. Second, the SNPs have no relationship to potential confounders. Third, IVs can only cause outcomes through exposure (29) (Figure 1).

### 2.2. Data sources

#### 2.2.1. GWAS summary data for PM and CVDs

GWAS summary statistics for PM were obtained from the UK Biobank, including about 423,796 participants from Europe, both male and female, which assessed the relationship between PM and SNPs. Briefly, this GWAS examined two PM phenotypes, including PM10 (*n* = 423,796) and PM2.5 (*n* = 423,796). In the case of AF (*n* = 103,083,6), we acquired summary-level data from the GWAS meta-analysis reported by Nielsen et al. (30), which included 60,620 AF cases and 970,216 controls and revealed 142 independent risk variants at 111 loci and prioritized 151 functional candidate genes likely to be involved in atrial fibrillation. MI (*n* = 395,795) was obtained from the GWAS conducted by Hartiala et al., which contained 395,795 participants from Europe, both male and female (31). HF (*n* = 977,323) was gained from the Heart Failure Molecular Epidemiology for Therapeutic Targets consortium (HERMES) conducted by Shah et al. (32). The GWAS comprised 47,309 HF cases and 930,014 controls, showing 12 independent variants at 11 genomic loci were associated with HF. IS (*n* = 440,328), large artery stroke (*n* = 150,765), small vessel stroke (*n* = 1988,048) and cardioembolic stroke (*n* = 211,763) was had access to the results conducted by Malik et al. (33), who tested 8 million SNPs in up to 67,162 stroke cases and 454,450 controls and revealed twenty-one additional loci were associated with stroke in this study. Descriptions of the datasets used in the analysis can be found in Table 1.

**Table 1:** Detailed information of studies and datasets used for analyses.

Phenotype	Data source	Sample size	Population	Web source
PM2.5	UK Biobank	423,796	European	https://gwas.mrcieu.ac.uk/datasets/ukb-b-10817/
PM10	UK Biobank	423,796	European	https://gwas.mrcieu.ac.uk/datasets/ukb-b-18469/
AF	Nielsen et al.	103,083,6	European	https://gwas.mrcieu.ac.uk/datasets/ebi-a-GCST006414/
HF	HERMES	977,323	European	https://gwas.mrcieu.ac.uk/datasets/ebi-a-GCST009541/
MI	Hartiala et al.	395,795	European	https://gwas.mrcieu.ac.uk/datasets/ebi-a-GCST011364/
IS	MEGASTROKE	440,328	European	https://gwas.mrcieu.ac.uk/datasets/ebi-a-GCST005843/
LAS	MEGASTROKE	150,765	European	https://gwas.mrcieu.ac.uk/datasets/ebi-a-GCST006907/
SVS	MEGASTROKE	198,048	European	https://gwas.mrcieu.ac.uk/datasets/ebi-a-GCST006909/
CS	MEGASTROKE	211,763	European	https://gwas.mrcieu.ac.uk/datasets/ebi-a-GCST006910/
BMI	Wood et al.	119,688	European	https://gwas.mrcieu.ac.uk/datasets/ebi-a-GCST006802/
TG	Within family GWAS consortium	78,700	European	https://gwas.mrcieu.ac.uk/datasets/ieu-b-4850/
HDL-C	Within family GWAS consortium	77,409	European	https://gwas.mrcieu.ac.uk/datasets/ieu-b-4844/
LDL-C	Within family GWAS consortium	70,814	European	https://gwas.mrcieu.ac.uk/datasets/ ieu-b-4846/
Fasting insulin	Chen et al.	151,013	European	https://gwas.mrcieu.ac.uk/datasets/ebi-a-GCST90002238/
Fasting glucose	Chen et al.	200,622	European	https://gwas.mrcieu.ac.uk/datasets/ebi-a-GCST90002232/
DBP	MRC-IEU	39,749	European	https://gwas.mrcieu.ac.uk/datasets/ukb-b-18240/
SBP	MRC-IEU	39,749	European	https://gwas.mrcieu.ac.uk/datasets/ukb-b-6503/

HERMES, heart failure molecular epidemiology for therapeutic targets; PM, particulate matter; AF, Atrial fibrillation; HF, Heart failure; MI, Myocardial infarction; IS, Ischemic stroke; LAS,Large artery stroke; SVS, Small vessel stroke; CS, Cardioembolic stroke;BMI, body mass index. TG, triglyceride; HDL-C, high-density lipoprotein cholesterol; LDL-C, low-density lipoprotein cholesterol; DBP, diastolic blood pressure; SBP, systolic blood pressure.

#### 2.2.2. GWAS summary data for cardiovascular biomarkers

The GWAS summary statistics of BMI were accessible to the study conducted by Wood et al., including 119,688 individuals from European (34). The GWAS summary statistics for lipid traits were available from the Within family GWAS consortium, containing TG, HDL-C, LDL-C, which included 78,700, 77,409, and 70,814 participants from Europe, both male and female. The GWAS summary statistics for fasting insulin and fasting glucose were obtained from the study conducted by Chen et al. (35). The GWAS summary statistics for blood pressure were accessible to the consortium of the MRC-IEU, including DBP and SBP. Descriptions of the datasets used in the analysis can be found in Table 1.

### 2.3. Selection and validation of SNPs

After we set the threshold of the *p* value as 5 × 10^−8^, we did not obtain any independent SNPs from the GWAS of PM10. In order to contain more SNPs that are concerned with PM10, we used a more lenient criterion (*p* < 5 × 10^−7^) which had been applied to previous MR research (36). Then we identified 16 SNPs connected with PM2.5 and 6 independent SNPs connected with PM10 at the genome-wide significance level (*p* < 5 × 10^−7^), showing the low likelihood of weak instrumental variable bias in MR analysis. No SNP was directly associated with CVDs or cardiovascular biomarkers. Using the TwoSampleMR R package, we conducted clumping functions in order to pick genetic variants without any linkage disequilibrium (LD) (*r*^2^ < 0.001 across a 10,000 kb window) (37). At last, 16 independent SNPs related to PM2.5 and 6 independent SNPs related to PM10 were determined; details can be found in the Supplementary Table. In addition, when the *F*-statistic is greater than 10, the SNPs were regarded as adequate to moderate the effect of potential bias, using the following formula: *F* = R^2^ × (N-2)/(1-R^2^) (38). No SNP was excluded from the MR analyses (The specific steps for selecting SNPs are shown in Figure 1).

### 2.4. MR analysis

This study used the two-sample MR method. As a primary analysis, we used inverse variance weighted (IVW) analyses under the fixed effects model as our main method because no heterogeneity was found in most analyses. Additionally, to ensure the results are robust, multiple complementary analyses were conducted like IVW under the random effects model, weighted median, and MR-egger. Sensitivity analyses like the MR-PRESSO test, Egger-intercept test, and the leave-one-out analysis are used to test whether the result of MR estimate is reliable. The Cochran Q test is mainly used to test and evaluate the heterogeneity of the selected IVs, which refers to the difference between the GWAS samples of exposure and outcome (39). We performed the MR-Egger intercept test in order to detect potential directional pleiotropy. It was determined that a significant pleiotropic bias existed when the intercept *p*-value <0.05 (40). We used the MR-PRESSO method to detect outliers before IVW methods were proceeded. MR-PRESSO eliminated abnormal SNPS (outliers) to detect potential horizontal pleiotropic and test whether there is a difference between the results before and after correction (41). The leave-one-out method was applied to analyze the sensitivity of the results by sequentially removing one SNP at a time to examine whether a single SNP with a large horizontal pleiotropy effect might affect the MR estimates. A total of MR analyses was performed using the R package “TwosampleMR.”

## 3. Result

### 3.1. Causal effect of PM and CVDs

**Figure 2:**
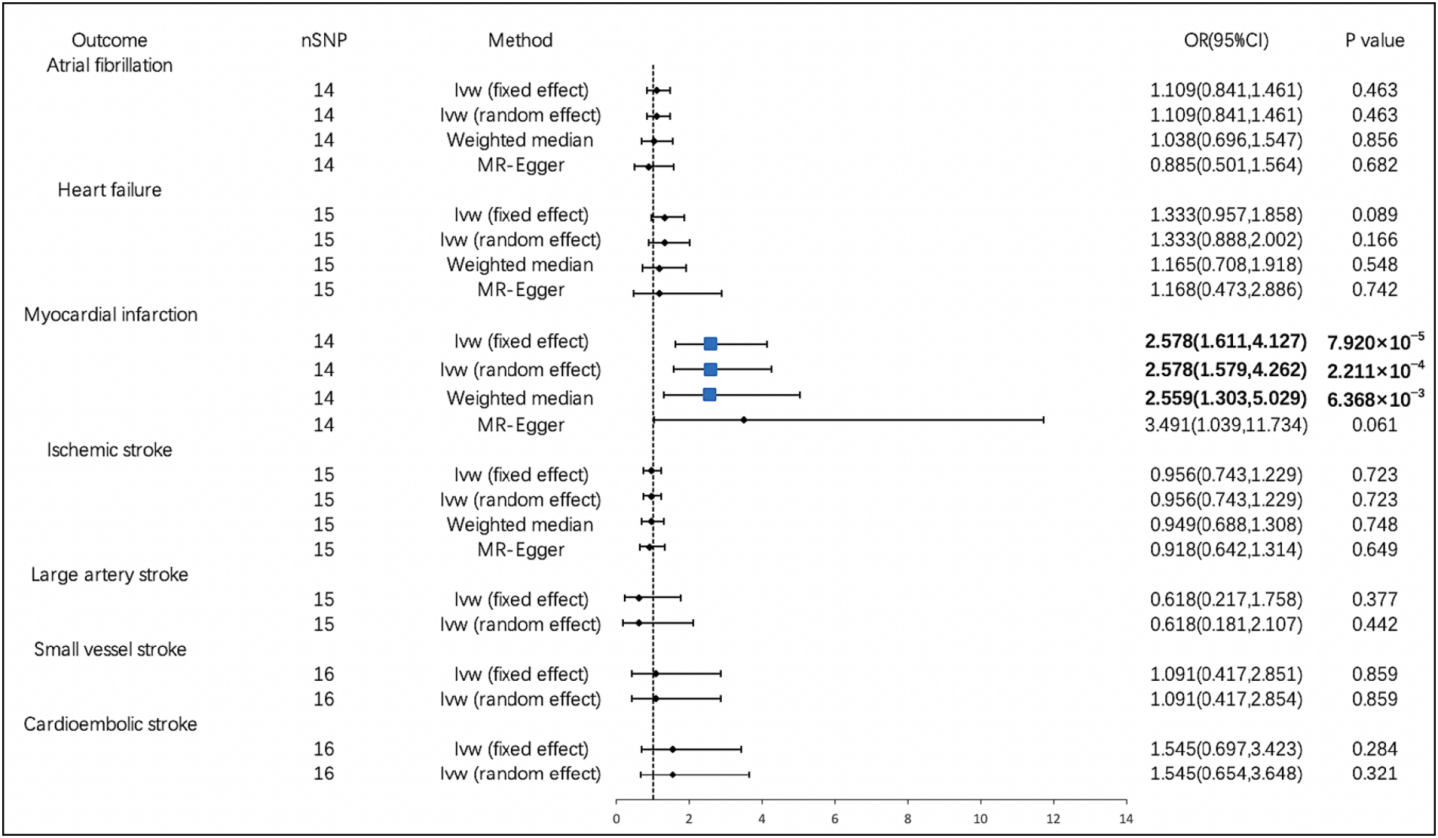
Associations of PM2.5 with cardiovascular diseases. CI, confidence interval; OR, odds ratio; SNP, single-nucleotide polymorphism.

**Figure 3:**
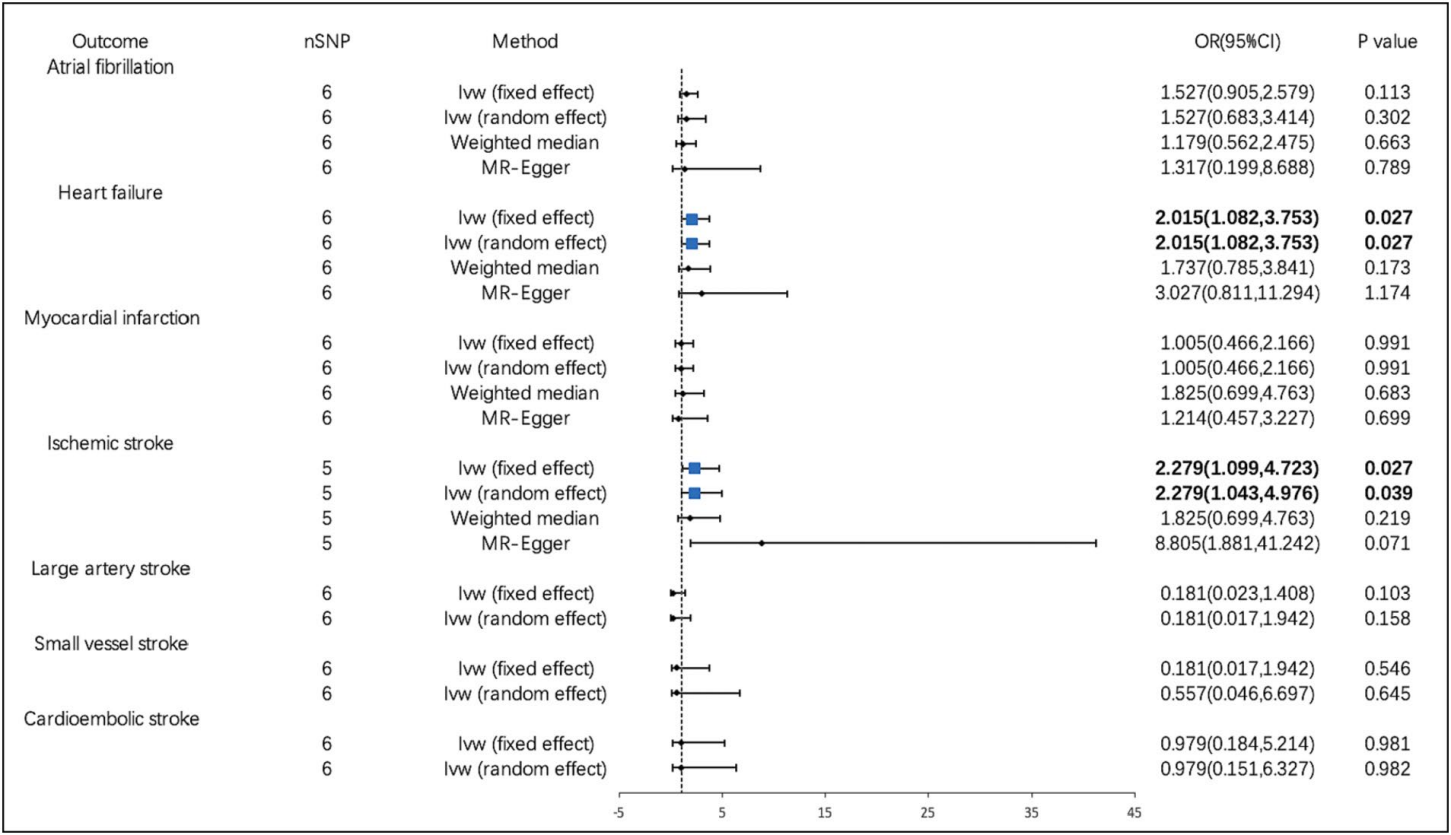
Associations of PM10 with cardiovascular diseases. CI, confidence interval; OR, odds ratio; SNP, single-nucleotide polymorphism.

According to the IVW analysis, PM2.5 concentration increases were significantly related to a higher risk of MI (OR, 2.578; 95% CI, 1.611–4.127; *p* = 7.920 × 10^−5^). Using the weighted median method, identical risk estimates were obtained as well (OR, 2.559; 95% CI, 1.303–5.029; *p* = 6.368 × 10^−3^) (Figure 2). Suggestive evidence was found between PM10 and HF (OR, 2.015; 95% CI, 1.082–3.753; *p* = 0.027) and IS (OR, 2.279; 95% CI, 1.099–4.723; p = 0.027) (Figure 3). In contrast, no causal relationship was found between PM2.5 and other CVDs (Figure 2). Furthermore, we did not observe the causality of PM10 on the risk of MI, AF, or stroke subtypes (Figure 3).

### 3.2. Causal effect of PM and cardiovascular biomarkers

**Figure 4:**
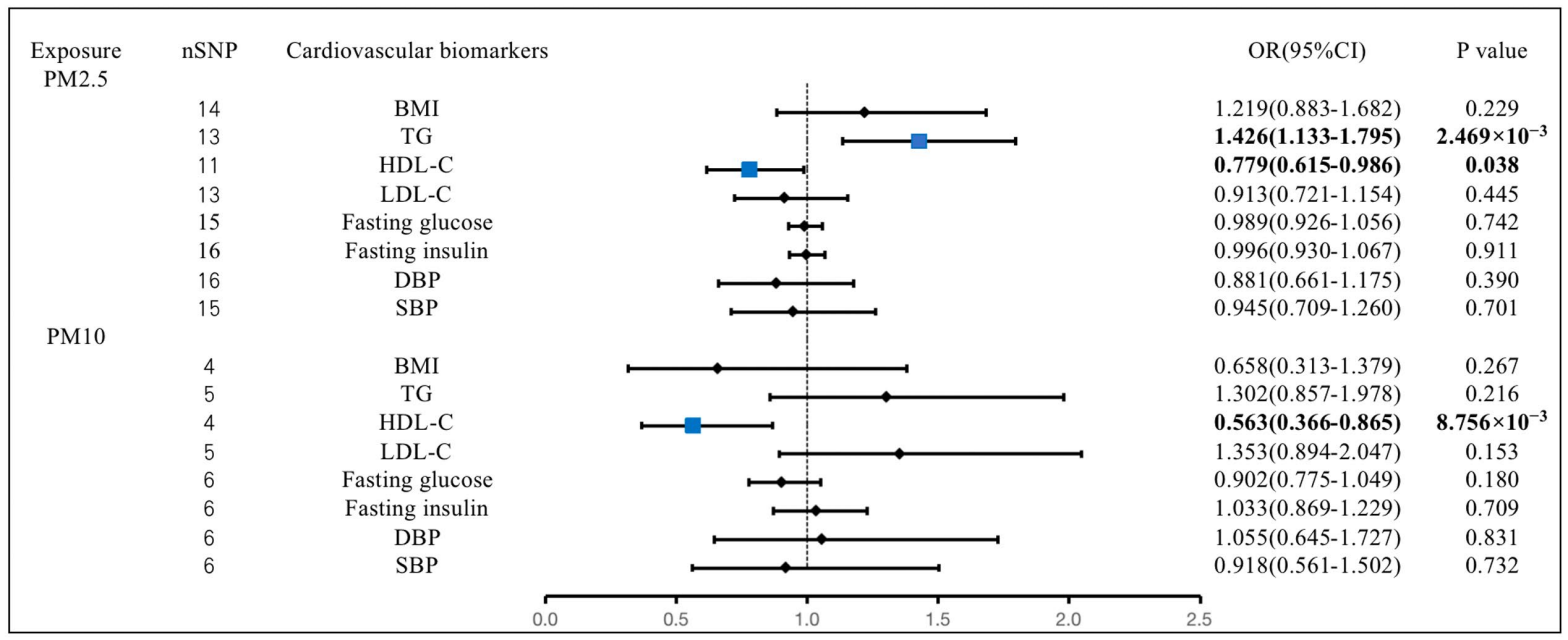
Associations of PM with cardiovascular biomarkers. CI, confidence interval; OR, odds ratio; SNP, single-nucleotide polymorphism. BMI, body mass index; TG, triglyceride; HDL-C, high-density lipoprotein cholesterol; LDL-C, low-density lipoprotein cholesterol; DBP, diastolic blood pressure; SBP, systolic blood pressure.

The IVW analysis revealed that the genetically predicted PM2.5 concentration increases were significantly associated with increases in TG (OR, 1.426; 95% CI, 1.133–1.795; *p* = 2.469 × 10^−3^). We also found that the increases in PM2.5 concentration were associated with the decreases in HDL-C (OR, 0.779; 95% CI, 0.615–0.986; *p* = 0.038). The PM10 concentration increases were also closely related to the decreases in HDL-C (OR, 0.563; 95% CI, 0.366–0.865; *p* = 8.756 × 10^−3^). We observed no causal effect of PM on BMI, LDL-C, fasting glucose, fasting insulin, DBP, or SBP. The results of the remaining methods can be found in Supplementary materials (Figure 4).

### 3.3. Sensitivity analysis

In sensitivity analysis, before we conducted the MR estimate, we used the method of MR-PRESSO to correct for the horizontal pleiotropy via outlier removal. After excluding these pleiotropic variants, no horizontal pleiotropy was found by conducted the MR-PRESSO method. For all outcomes, according to the MR-Egger regression, there did not appear to be horizontal pleiotropy based on the intercept term (Pintercept >0.05). We used the leave-one-out study to test the robustness of the results. All error lines are to the left of 0, indicating that the results are reliable and demonstrating that no SNPs with a large horizontal pleiotropic effect. We found modest heterogeneity in several analyses. However, heterogeneity did not affect the reliability of our conclusion. Plots of the leave-one-out analyses were also exhibited in Supplementary Figures. Details of the sensitivity analyses can be found in Tables 2–5.

**Table 2:** Sensitive analyses for the Mendelian randomization analysis between PM 2.5 and cardiovascular diseases.

Outcomes	Pleiotropy test (outliers-corrected)		Heterogeneity test (outliers-corrected)			Outliers
	MR-PRESSO global test *p* value	MR-Egger intercept test *p* value	Cochran’s Q	Degrees of freedom	Cochran’s Q *p* value	
AF	0.899	0.393	7.471	13	0.876	rs114708313 rs1537371
HF	0.135	0.752	19.542	13	0.107	rs1537371
MI	0.385	0.598	14.843	13	0.317	rs12203592 rs1537371
IS	0.748	0.766	11.382	14	0.656	rs1537371
LAS	0.127	0.095	19.266	14	0.155	rs1537371
SVS	0.432	0.619	15.036	15	0.449	NA
CS	0.303	0.149	17.497	15	0.291	NA

AF, Atrial fibrillation; HF, Heart failure; MI, Myocardial infarction; IS, Ischemic stroke; LAS,Large artery stroke; SVS, Small vessel stroke; CS, Cardioembolic stroke.

**Table 3:** Sensitive analyses for the Mendelian randomization analysis between PM 10 and cardiovascular diseases.

Outcomes	Pleiotropy test (outliers-corrected)		Heterogeneity test (outliers-corrected)			Outliers
	MR-PRESSO global test *p* value	MR-Egger intercept test *p* value	Cochran’s Q	Degrees of Freedom	Cochran’s Q *p* value	
AF	0.063	0.869	11.796	5	0.038	NA
HF	0.594	0.529	3.949	5	0.557	NA
MI	0.758	0.657	2.711	5	0.744	NA
IS	0.374	0.147	4.594	4	0.332	NA
LAS	0.275	0.693	6.689	5	0.245	NA
SVS	0.167	0.760	8.591	5	0.127	NA
CS	0.311	0.819	6.224	5	0.285	NA

AF, Atrial fibrillation; HF, Heart failure; MI, Myocardial infarction; IS, Ischemic stroke; LAS,Large artery stroke; SVS, Small vessel stroke; CS, Cardioembolic stroke.

**Table 4:** Sensitive analyses for the Mendelian randomization analysis between PM 2.5 and cardiovascular biomarkers.

Outcomes	Pleiotropy test (outliers-corrected)		Heterogeneity test (outliers-corrected)			Outliers
	MR-PRESSO global test *p* value	MR-Egger intercept test *p* value	Cochran’s Q	Degrees of freedom	Cochran’s Q *p* value	
BMI	0.102	0.507	19.611	13	0.086	NA
TG	0.326	0.894	13.935	12	0.305	NA
HDL-C	0.947	0.386	4.079	10	0.944	rs72808024 rs1318845
LDL-C	0.018	0.111	24.691	12	0.016	NA
Fasting insulin	0.069	0.926	26.547	15	0.033	NA
Fasting glucose	0.660	0.630	12.072	14	0.601	NA
DBP	0.425	0.586	14.407	14	0.420	NA
SBP	0.928	0.472	7.123	14	0.930	NA

BMI, body mass index. TG, triglyceride; HDL-C, high-density lipoprotein cholesterol; LDL-C, low-density lipoprotein cholesterol; DBP, diastolic blood pressure; SBP, systolic blood pressure.

**Table 5:** Sensitive analyses for the Mendelian randomization analysis between PM 10 and cardiovascular biomarkers.

Outcomes	Pleiotropy test (outliers-corrected)		Heterogeneity test (outliers-corrected)			Outliers
	MR-PRESSO global test *p* value	MR-Egger intercept test *p* value	Cochran’s Q	Degrees of Freedom	Cochran’s Q *p* value	
BMI	0.457	0.771	3.790	3	0.285	NA
TG	0.945	0.523	0.751	4	0.945	NA
HDL-C	0.435	0.332	3.153	3	0.369	rs117671171
LDL-C	0.357	0.822	4.901	4	0.298	NA
Fasting insulin	0.269	0.856	6.552	5	0.256	NA
Fasting glucose	0.952	0.486	1.078	5	0.956	NA
DBP	0.858	0.597	1.986	5	0.851	NA
SBP	0.448	0.649	5.100	5	0.404	NA

BMI, body mass index. TG, triglyceride; HDL-C, high-density lipoprotein cholesterol; LDL-C, low-density lipoprotein cholesterol; DBP, diastolic blood pressure; SBP, systolic blood pressure.

## 4. Discussion

In the present MR study, we examined the causality of PM on CVDs and cardiovascular biomarkers. Our findings showed PM2.5 was significantly related to a higher risk of MI and revealed a suggestive causal association between PM10 and the risk of HF and IS, which might be explained by deleterious effects on blood lipid levels.

With the increasing abundance of GWAS data, many scholars conducted MR analysis to infer causality between PM2.5 and diseases (42, 43). Zhang et al. identified 7 SNPs related to PM2.5 and conducted two sample MR methods to conclude that PM2.5 concentrations can increase the risk of hypothyroidism (43). Yang et al. used 85 SNPs as genetic variants for PM2.5 and demonstrated a causal relationship between PM2.5 and gestational diabetes mellitus (42). Our research is the first MR Study focusing on the causal effect of PM on CVDs and cardiovascular biomarkers. Previous analyses have shown that exposure to PM is associated with an increased risk of MI, IS, and HF (44). To identify whether PM2.5 contributes to a rise in the incidence of MI, Li et al. carried out a meta-analysis that comprised twenty-seven cohort studies involving 6,764,987 participants and 94,540 patients with MI. The analysis concluded that long-term exposure to PM2.5 plays a role in MI (45). A meta-analysis by Li et al., which included 27 cohort studies involving more than 6.5 million people, showed a positive association between exposure to PM2.5 and the risk of MI (45). Farhadi and his colleagues noted the severity of the relationship between PM2.5 and MI in a meta-analysis of 26 studies, confirming the notion that PM2.5 levels are a key factor in the development of MI hospitalization (15). Studies have confirmed that air pollutants can trigger oxidative stress and inflammation in the body through direct entry into the blood or indirectly through particles, metals, and other components in the substance, leading to vasoconstriction, endothelial dysfunction, platelet hyperresponsiveness, and even autonomic nervous system disorders, thus increasing the risk of CVDs (46–48).

A meta-analysis of 35 studies showed that exposure to PM pollution was associated with an increased risk of hospitalization or death from HF. For every 10 μg/m^3^ increase in exposure to PM10, the risk of hospitalization or death from HF increased by 1.6% (49). A study of more than 100,000 hospitalizations for HF from 26 major cities in China analyzed the association between various air pollutants and hospitalizations for HF. The concentration of PM10 increased by one quartile (76.9 μg/m^3^), the risk of hospitalization for HF increased by 1.3% (50). As Brook and his colleagues reported, PM has been linked with higher systemic blood pressure and vasoconstriction (51). Exposure to PM increases right ventricular and pulmonary diastolic filling pressures (52). Air pollution will dramatically raise the requirements based on the failing heart, possibly precipitating acute decompensation.

Our results concerning the potential causal relationship between PM10 and the risk of IS cohere with a systematic review that included 94 studies from 28 countries that reported that the short-run exposure concentration of PM10 increased by 10 μg/m^3^, the risk of hospitalization and death from stroke increased by 0.3%. On the other hand, Hossein et al. expressed that the level of PM was immediately connected with the number of stroke inpatients in the emergency room. Long-run changes in PM10 also increased the risk of IS (53). Research has speculated that increased blood pressure triggered by acute increases in pollutant concentrations can contribute to the development of IS (54, 55).

In terms of cardiovascular biomarkers, our study showed that PM2.5 concentration increases were significantly associated with increases in TG and decreases in HDL-C. The PM10 concentration increases were also closely related to the decreases in HDL-C. Information concerning the relationship between PM and changes in lipids and lipoproteins was disputable, especially regarding HDL-C (56–59). Multiple studies have demonstrated an inverse relationship between high concentrations of PM exposure and the level of HDL-C (60–62). Zhang et al. revealed that PM10 was negatively associated with HDL-C (63). A study conducted by Wang et al. showed that each increase in PM2.5 was associated with higher levels of TG and lower levels of HDL-C (64). A health study involving 33 communities concluded that per 10-μg/m3 increment in PM2.5 was significantly associated with 1.1% (95% CI: 0.4, 1.8%) increases in TG and 1.1% (95% CI: 0.8, 1.4%) decreases in HDL-C (65). Consistently, a longitudinal study performed in a multiethnic US cohort free of cardiovascular disease showed that higher exposure to PM2.5 was associated with lower HDL particle numbers or lower HDL-C levels (57). The precise mechanisms between PM exposures and lipid metabolism have not been fully determined. Several studies have shown that exposure to air pollution can lead to adverse lipid metabolism and lipid oxidation through systemic inflammation and oxidative stress (66–68). Other studies suggested that ambient air pollution might cause DNA methylation, leading to changes in particular genes associated with lipid metabolism (69, 70). Larger and more controlled studies are needed to fully address this issue.

### 4.1. Limitations and strengths

The advantages of our research are as follows. Our research is the first MR study focusing on the effect of PM on CVDs and cardiovascular biomarkers. MR estimates can avoid the interference of confounding factors and reveal the causal relationship between PM and CVDs more confidently. MR estimates can avoid the interference of confounding factors and reverse causality compared with other observational studies. However, this study also has some limitations. Firstly, the GWAS of exposure failed to distinguish the time of PM pollution, so our results cannot explain the time of exposure effect, which limited us from conducting a further analysis. Several studies have also proposed a difference between “long-term” and “short-term” outcomes. Short-term exposure to air pollution is thought to cause acute CVDs events by destabilizing susceptible plaques, although long-term exposure may increase the risk of atherosclerosis by accelerating dyslipidemia, hypertension, and other metabolic disturbances (71). The future GWAS study of PM needs to distinguish the duration of exposure. Secondly, when we selected IVs, we used a more lenient threshold (*p* < 5 × 10^−7^). Although this may boost statistical power, the more instrumental variables included in the study, the greater the possibility of producing more pleiotropy. In order to eliminate horizontal pleiotropy, we conducted MR sensitive analyses such as the MR-Egger intercept, MR-PRESSO, and leave-one-out method. However, it is very difficult to completely exclude directional pleiotropy because SNPs affect exposure and outcome through unknown pathways, which has decreased the reliability of the findings. At last, the GWAS selected in this study are from European populations, and whether the findings of this study are applicable to other populations remains to be determined. In particular, there are regional differences in PM pollution, especially in some developing countries, but Europe, the main body of this study, is mostly developed countries. Therefore, our results could not be easily generalized to populations in high-pollution areas. Finally, there was modest sample overlap between the GWAS of PM and the GWAS of AF, HF, MI, SBP, and DBP, which might result in bias. Nevertheless, the F-statistic was large enough to moderate the effect of potential bias by sample overlap (72). Moreover, if the genetic link with PM was solely evaluated in non-cases, this sample overlap would not result in bias.

### 4.2. Perspectives and future research

Several epidemiological and experimental studies have regarded all components of PM as associated risk factors for the occurrence and deterioration of cardiovascular disease, which is independent of other conventional risk factors, including smoking, obesity, and diabetes. Our findings demonstrated the different effects of PM10 and PM2.5 on CVDs and cardiovascular biomarkers. Despite many studies suggesting that smaller size fractions are more harmful, especially PM2.5 (73, 74), the risks to human health posed by coarse particles like PM10 cannot be ignored. It was reported that PM10 contained more lipopolysaccharide (LPS) than PM2.5, which could trigger inflammation by directly activating Toll-like receptors and induce metabolic syndromes (75–77). Furthermore, a study conducted by Chang et al. suggested that heart rate variability (HRV) changes are reliant on PM10 as opposed to smaller particles (78). It is urgent for us to conduct more research to identify the nature of PM in all its size fractions and investigate the molecular mechanisms underlying the effects of PM with different sizes on the CVDs. Randomized controlled trials on this topic should be conducted in the future, although this is highly challenging. Besides, a handful of studies have shown that higher levels of PM impair the function of HDL (60, 79, 80), which might affect the development of CVDs beyond possible changes in plasma HDL-C levels. Larger and more controlled studies are needed to investigate the mechanisms by which HDL functionality and levels are affected by PM exposure and explore the potential efficiency of preventative interventions like statins and antioxidant therapy. To protect the earth together, we hope that all countries will strengthen the effective control of pollution sources, adopt strict law enforcement and management measures, and pay attention to the efficient combination of policy and scientific and technological innovation.

## 5. Conclusion

At the genetic level, our study provides evidence supporting the causality of PM on CVDs and cardiovascular biomarkers. Regarding CVDs, PM2.5 concentration increases were significantly associated with a higher risk of MI. PM10 concentration increases were significantly associated with a higher risk of HF and IS. In terms of cardiovascular biomarkers, PM2.5 concentration increases were significantly correlated with increases in TG and decreases in HDL-C. The PM10 concentration increases were also closely correlated with the decreases in HDL-C. The underlying pathophysiological mechanisms need to be further studied. Therefore, more research should be carried out to explore the mechanism and prevention of PM exposure in CVDs to better contribute to people’s healthy lives.

## Data availability statement

The original contributions presented in the study are included in the article/Supplementary material, further inquiries can be directed to the corresponding authors.

## Author contributions

QW came up with the concept and designed the study and prepared the manuscript. ZW, MC, WM, and ZX processed the data. MX polished the draft. All authors contributed to the article and approved the submitted version.

## Conflict of interest

The authors declare that the research was conducted in the absence of any commercial or financial relationships that could be construed as a potential conflict of interest.

## Publisher’s note

All claims expressed in this article are solely those of the authors and do not necessarily represent those of their affiliated organizations, or those of the publisher, the editors and the reviewers. Any product that may be evaluated in this article, or claim that may be made by its manufacturer, is not guaranteed or endorsed by the publisher.

## References

[1] Roth GA Johnson C Abajobir A Abd-Allah F Abera SF Abyu G. Global, regional, and National Burden of cardiovascular diseases for 10 causes, 1990 to 2015. J Am Coll Cardiol. (2017) 70:1–25. doi: 10.1016/j.jacc.2017.04.052, PMID: 28527533 PMC5491406

[2] Burnett R Chen H Szyszkowicz M Fann N Hubbell B Pope CA 3rd. Global estimates of mortality associated with long-term exposure to outdoor fine particulate matter. Proc Natl Acad Sci U S A. (2018) 115:9592–7. doi: 10.1073/pnas.1803222115, PMID: 30181279 PMC6156628

[3] Miller MR Shaw CA Langrish JP. From particles to patients: oxidative stress and the cardiovascular effects of air pollution. Futur Cardiol. (2012) 8:577–602. doi: 10.2217/fca.12.43, PMID: 22871197

[4] Faraji Ghasemi F Dobaradaran S Saeedi R Nabipour I Nazmara S Abadi DRV. Levels and ecological and health risk assessment of PM(2.5)-bound heavy metals in the northern part of the Persian Gulf. Environ Sci Pollut Res Int. (2020) 27:5305–13. doi: 10.1007/s11356-019-07272-7 31848967

[5] Goudarzi G Alavi N Geravandi S Idani E Behrooz HRA Babaei AA. Health risk assessment on human exposed to heavy metals in the ambient air PM(10) in Ahvaz, Southwest Iran. Int J Biometeorol. (2018) 62:1075–83. doi: 10.1007/s00484-018-1510-x, PMID: 29464337

[6] Abbasi-Kangevari M Malekpour MR Masinaei M Moghaddam SS Ghamari SH Abbasi-Kangevari Z. Effect of air pollution on disease burden, mortality, and life expectancy in North Africa and the Middle East: a systematic analysis for the global burden of disease study 2019. Lancet Planet Health. (2023) 7:e358–69. doi: 10.1016/S2542-5196(23)00053-0, PMID: 37164512 PMC10186179

[7] Xing YF Xu YH Shi MH Lian YX. The impact of PM2.5 on the human respiratory system. J Thorac Dis. (2016) 8:E69–74. doi: 10.3978/j.issn.2072-1439.2016.01.19, PMID: 26904255 PMC4740125

[8] Pope CA 3rd Dockery DW. Health effects of fine particulate air pollution: lines that connect. J Air Waste Manag Assoc. (2006) 56:709–42. doi: 10.1080/10473289.2006.10464485, PMID: 16805397

[9] Brook RD Franklin B Cascio W Hong Y Howard G Lipsett M. Air pollution and cardiovascular disease: a statement for healthcare professionals from the expert panel on population and prevention science of the American Heart Association. Circulation. (2004) 109:2655–71. doi: 10.1161/01.CIR.0000128587.30041.C8 15173049

[10] Borsi SH Goudarzi G Sarizadeh G Dastoorpoor M Geravandi S Shahriyari HA. Health endpoint of exposure to criteria air pollutants in ambient air of on a populated in Ahvaz City, Iran. Front Public Health. (2022) 10:869656. doi: 10.3389/fpubh.2022.869656, PMID: 35425736 PMC9002232

[11] Dastoorpoor M Sekhavatpour Z Masoumi K Mohammadi MJ Aghababaeian H Khanjani N. Air pollution and hospital admissions for cardiovascular diseases in Ahvaz, Iran. Sci Total Environ. (2019) 652:1318–30. doi: 10.1016/j.scitotenv.2018.10.285, PMID: 30586817

[12] Moradi M Mokhtari A Mohammadi MJ Hadei M Vosoughi M. Estimation of long-term and short-term health effects attributed to PM(2.5) standard pollutants in the air of Ardabil (using air Q + model). Environ Sci Pollut Res Int. (2022) 29:21508–16. doi: 10.1007/s11356-021-17303-x, PMID: 34761318

[13] Shao Q Liu T Korantzopoulos P Zhang Z Zhao J Li G. Association between air pollution and development of atrial fibrillation: a meta-analysis of observational studies. Heart Lung. (2016) 45:557–62. doi: 10.1016/j.hrtlng.2016.08.001, PMID: 27590407

[14] Yang C Chen A Chen R Qi Y Ye J Li S. Acute effect of ambient air pollution on heart failure in Guangzhou, China. Int J Cardiol. (2014) 177:436–41. doi: 10.1016/j.ijcard.2014.09.003, PMID: 25442978

[15] Farhadi Z Abulghasem Gorgi H Shabaninejad H Aghajani Delavar M Torani S. Association between PM2.5 and risk of hospitalization for myocardial infarction: a systematic review and a meta-analysis. BMC Public Health. (2020) 20:314. doi: 10.1186/s12889-020-8262-3, PMID: 32164596 PMC7068986

[16] Yang WS Wang X Deng Q Fan WY Wang WY. An evidence-based appraisal of global association between air pollution and risk of stroke. Int J Cardiol. (2014) 175:307–13. doi: 10.1016/j.ijcard.2014.05.044 24866079

[17] Pope CA 3rd Muhlestein JB May HT Renlund DG Anderson JL Horne BD. Ischemic heart disease events triggered by short-term exposure to fine particulate air pollution. Circulation. (2006) 114:2443–8. doi: 10.1161/CIRCULATIONAHA.106.636977, PMID: 17101851

[18] Cramer J Jørgensen JT Hoffmann B Loft S Bräuner EV Prescott E. Long-term exposure to air pollution and incidence of myocardial infarction: a Danish nurse cohort study. Environ Health Perspect. (2020) 128:57003. doi: 10.1289/EHP5818, PMID: 32438827 PMC7263451

[19] O'Neill MS Diez-Roux AV Auchincloss AH Shen M Lima JA Polak JF. Long-term exposure to airborne particles and arterial stiffness: the multi-ethnic study of atherosclerosis (MESA). Environ Health Perspect. (2011) 119:844–51. doi: 10.1289/ehp.0901524, PMID: 21245016 PMC3114821

[20] Barnett AG Williams GM Schwartz J Best TL Neller AH Petroeschevsky AL. The effects of air pollution on hospitalizations for cardiovascular disease in elderly people in Australian and New Zealand cities. Environ Health Perspect. (2006) 114:1018–23. doi: 10.1289/ehp.8674, PMID: 16835053 PMC1513338

[21] Mills NL Robinson SD Fokkens PH Leseman DL Miller MR Anderson D. Exposure to concentrated ambient particles does not affect vascular function in patients with coronary heart disease. Environ Health Perspect. (2008) 116:709–15. doi: 10.1289/ehp.11016, PMID: 18560524 PMC2430224

[22] Madaniyazi L Li S Li S Guo Y. Candidate gene expression in response to low-level air pollution. Environ Int. (2020) 140:105610. doi: 10.1016/j.envint.2020.105610, PMID: 32248990

[23] Zhou Z Qin M Khodahemmati S Li W Niu B Li J. Gene expression in human umbilical vein endothelial cells exposed to fine particulate matter: RNA sequencing analysis. Int J Environ Health Res. (2022) 32:2052–64. doi: 10.1080/09603123.2021.1935785, PMID: 34102927

[24] Jung I Park M Jeong MH Park K Kim WH Kim GY. Transcriptional analysis of gasoline engine exhaust particulate matter 2.5-exposed human umbilical vein endothelial cells reveals the different gene expression patterns related to the cardiovascular diseases. Biochem Biophys Rep. (2022) 29:101190. doi: 10.1016/j.bbrep.2021.101190 34988296 PMC8695280

[25] Yao Y Liu L Guo G Zeng Y Ji JS. Interaction of Sirtuin 1 (SIRT1) candidate longevity gene and particulate matter (PM2.5) on all-cause mortality: a longitudinal cohort study in China. Environ Health. (2021) 20:25. doi: 10.1186/s12940-021-00718-x, PMID: 33715628 PMC7958462

[26] Poursafa P Kelishadi R Haghjooy-Javanmard S Rafiei L Keramatian K. Synergistic effects of genetic polymorphism and air pollution on markers of endothelial dysfunction in children. J Res Med Sci. (2012) 17:718–23. PMID: 23798936 PMC3687876

[27] Smith GD Ebrahim S. Mendelian randomization: prospects, potentials, and limitations. Int J Epidemiol. (2004) 33:30–42. doi: 10.1093/ije/dyh132, PMID: 15075143

[28] Lawlor DA Harbord RM Sterne JA Timpson N Davey Smith G. Mendelian randomization: using genes as instruments for making causal inferences in epidemiology. Stat Med. (2008) 27:1133–63. doi: 10.1002/sim.3034 17886233

[29] Burgess S Scott RA Timpson NJ Davey Smith G Thompson SG. Using published data in Mendelian randomization: a blueprint for efficient identification of causal risk factors. Eur J Epidemiol. (2015) 30:543–52. doi: 10.1007/s10654-015-0011-z, PMID: 25773750 PMC4516908

[30] Nielsen JB Thorolfsdottir RB Fritsche LG Zhou W Skov MW Graham SE. Biobank-driven genomic discovery yields new insight into atrial fibrillation biology. Nat Genet. (2018) 50:1234–9. doi: 10.1038/s41588-018-0171-3, PMID: 30061737 PMC6530775

[31] Hartiala JA Han Y Jia Q Hilser JR Huang P Gukasyan J. Genome-wide analysis identifies novel susceptibility loci for myocardial infarction. Eur Heart J. (2021) 42:919–33. doi: 10.1093/eurheartj/ehaa1040, PMID: 33532862 PMC7936531

[32] Shah S Henry A Roselli C Lin H Sveinbjörnsson G Fatemifar G. Genome-wide association and Mendelian randomisation analysis provide insights into the pathogenesis of heart failure. Nat Commun. (2020) 11:163. doi: 10.1038/s41467-019-13690-5, PMID: 31919418 PMC6952380

[33] Malik R Chauhan G Traylor M Sargurupremraj M Okada Y Mishra A. Multiancestry genome-wide association study of 520,000 subjects identifies 32 loci associated with stroke and stroke subtypes. Nat Genet. (2018) 50:524–37. doi: 10.1038/s41588-018-0058-3, PMID: 29531354 PMC5968830

[34] Wood AR Tyrrell J Beaumont R Jones SE Tuke MA Ruth KS. Variants in the FTO and CDKAL1 loci have recessive effects on risk of obesity and type 2 diabetes, respectively. Diabetologia. (2016) 59:1214–21. doi: 10.1007/s00125-016-3908-5, PMID: 26961502 PMC4869698

[35] Chen J Spracklen CN Marenne G Varshney A Corbin LJ Luan J. The trans-ancestral genomic architecture of glycemic traits. Nat Genet. (2021) 53:840–60. doi: 10.1038/s41588-021-00852-9, PMID: 34059833 PMC7610958

[36] Ong JS MacGregor S. Implementing MR-PRESSO and GCTA-GSMR for pleiotropy assessment in Mendelian randomization studies from a practitioner's perspective. Genet Epidemiol. (2019) 43:609–16. doi: 10.1002/gepi.22207, PMID: 31045282 PMC6767464

[37] Auton A Brooks LD Durbin RM Garrison EP Kang HM Korbel JO. A global reference for human genetic variation. Nature. (2015) 526:68–74. doi: 10.1038/nature15393, PMID: 26432245 PMC4750478

[38] Burgess S Thompson SG. Avoiding bias from weak instruments in Mendelian randomization studies. Int J Epidemiol. (2011) 40:755–64. doi: 10.1093/ije/dyr036, PMID: 21414999

[39] Greco MF Minelli C Sheehan NA Thompson JR. Detecting pleiotropy in Mendelian randomisation studies with summary data and a continuous outcome. Stat Med. (2015) 34:2926–40. doi: 10.1002/sim.6522 25950993

[40] Bowden J Davey Smith G Burgess S. Mendelian randomization with invalid instruments: effect estimation and bias detection through egger regression. Int J Epidemiol. (2015) 44:512–25. doi: 10.1093/ije/dyv080, PMID: 26050253 PMC4469799

[41] Verbanck M Chen CY Neale B Do R. Detection of widespread horizontal pleiotropy in causal relationships inferred from Mendelian randomization between complex traits and diseases. Nat Genet. (2018) 50:693–8. doi: 10.1038/s41588-018-0099-7, PMID: 29686387 PMC6083837

[42] Yang Y Ma X Pang W Jiang C. Causal associations of PM2.5 and GDM: a two-sample Mendelian randomization study. Toxics. (2023) 11:171. doi: 10.3390/toxics11020171 36851046 PMC9961059

[43] Zhang Y Liu S Wang Y Wang Y. Causal relationship between particulate matter 2.5 and hypothyroidism: a two-sample Mendelian randomization study. Front. Public Health. (2022) 10:1000103. doi: 10.3389/fpubh.2022.1000103 PMC9732245 36504957

[44] Bourdrel T Bind MA Béjot Y Morel O Argacha JF. Cardiovascular effects of air pollution. Arch Cardiovasc Dis. (2017) 110:634–42. doi: 10.1016/j.acvd.2017.05.003, PMID: 28735838 PMC5963518

[45] Zou L Zong Q Fu W Zhang Z Xu H Yan S. Long-term exposure to ambient air pollution and myocardial infarction: a systematic review and Meta-analysis. Front Med (Lausanne). (2021) 8:616355. doi: 10.3389/fmed.2021.616355, PMID: 33816520 PMC8010182

[46] Fiordelisi A Piscitelli P Trimarco B Coscioni E Iaccarino G Sorriento D. The mechanisms of air pollution and particulate matter in cardiovascular diseases. Heart Fail Rev. (2017) 22:337–47. doi: 10.1007/s10741-017-9606-7 28303426

[47] McGuinn LA Schneider A McGarrah RW Ward-Caviness C Neas LM Di Q. Association of long-term PM(2.5) exposure with traditional and novel lipid measures related to cardiovascular disease risk. Environ Int. (2019) 122:193–200. doi: 10.1016/j.envint.2018.11.001, PMID: 30446244 PMC6467069

[48] Choi YJ Kim SH Kang SH Kim SY Kim OJ Yoon CH. Short-term effects of air pollution on blood pressure. Sci Rep. (2019) 9:20298. doi: 10.1038/s41598-019-56413-y, PMID: 31889065 PMC6937254

[49] Shah AS Langrish JP Nair H McAllister DA Hunter AL Donaldson K. Global association of air pollution and heart failure: a systematic review and meta-analysis. Lancet. (2013) 382:1039–48. doi: 10.1016/S0140-6736(13)60898-3, PMID: 23849322 PMC3809511

[50] Liu H Tian Y Song J Cao Y Xiang X Huang C. Effect of ambient air pollution on hospitalization for heart failure in 26 of China's largest cities. Am J Cardiol. (2018) 121:628–33. doi: 10.1016/j.amjcard.2017.11.039, PMID: 29304993

[51] Brook RD Brook JR Urch B Vincent R Rajagopalan S Silverman F. Inhalation of fine particulate air pollution and ozone causes acute arterial vasoconstriction in healthy adults. Circulation. (2002) 105:1534–6. doi: 10.1161/01.CIR.0000013838.94747.64, PMID: 11927516

[52] Rich DQ Freudenberger RS Ohman-Strickland P Cho Y Kipen HM. Right heart pressure increases after acute increases in ambient particulate concentration. Environ Health Perspect. (2008) 116:1167–71. doi: 10.1289/ehp.11230, PMID: 18795158 PMC2535617

[53] Alimohammadi H Fakhri S Derakhshanfar H Hosseini-Zijoud SM Safari S Hatamabadi HR. The effects of air pollution on ischemic stroke admission rate. Chonnam Med J. (2016) 52:53–8. doi: 10.4068/cmj.2016.52.1.53, PMID: 26866000 PMC4742610

[54] Brook RD Newby DE Rajagopalan S. Air pollution and Cardiometabolic disease: an update and call for clinical trials. Am J Hypertens. (2017) 31:1–10. doi: 10.1093/ajh/hpx109, PMID: 28655143 PMC5861586

[55] Giorgini P Di Giosia P Grassi D Rubenfire M Brook RD Ferri C. Air pollution exposure and blood pressure: an updated review of the literature. Curr Pharm Des. (2016) 22:28–51. doi: 10.2174/1381612822666151109111712, PMID: 26548310

[56] Wu XM Broadwin R Basu R Malig B Ebisu K Gold EB. Associations between fine particulate matter and changes in lipids/lipoproteins among midlife women. Sci Total Environ. (2019) 654:1179–86. doi: 10.1016/j.scitotenv.2018.11.149, PMID: 30841392 PMC6413864

[57] Bell G Mora S Greenland P Tsai M Gill E Kaufman JD. Association of air Pollution Exposures with High-Density Lipoprotein Cholesterol and Particle Number: the multi-ethnic study of atherosclerosis. Arterioscler Thromb Vasc Biol. (2017) 37:976–82. doi: 10.1161/ATVBAHA.116.308193, PMID: 28408373 PMC5407952

[58] Fioravanti S Cesaroni G Badaloni C Michelozzi P Forastiere F Porta D. Traffic-related air pollution and childhood obesity in an Italian birth cohort. Environ Res. (2018) 160:479–86. doi: 10.1016/j.envres.2017.10.003 29078141

[59] Gui ZH Yang BY Zou ZY Ma J Jing J Wang HJ. Exposure to ambient air pollution and blood lipids in children and adolescents: a national population based study in China. Environ Pollut. (2020) 266:115422. doi: 10.1016/j.envpol.2020.115422, PMID: 32829032

[60] Li J Zhou C Xu H Brook RD Liu S Yi T. Ambient air pollution is associated with HDL (high-density lipoprotein) dysfunction in healthy adults. Arterioscler Thromb Vasc Biol. (2019) 39:513–22. doi: 10.1161/ATVBAHA.118.311749, PMID: 30700134

[61] Li R Navab M Pakbin P Ning Z Navab K Hough G. Ambient ultrafine particles alter lipid metabolism and HDL anti-oxidant capacity in LDLR-null mice. J Lipid Res. (2013) 54:1608–15. doi: 10.1194/jlr.M035014, PMID: 23564731 PMC3646462

[62] Chuang KJ Yan YH Chiu SY Cheng TJ. Long-term air pollution exposure and risk factors for cardiovascular diseases among the elderly in Taiwan. Occup Environ Med. (2011) 68:64–8. doi: 10.1136/oem.2009.052704, PMID: 20833756

[63] Zhang K Wang H He W Chen G Lu P Xu R. The association between ambient air pollution and blood lipids: a longitudinal study in Shijiazhuang, China. Sci Total Environ. (2021) 752:141648. doi: 10.1016/j.scitotenv.2020.141648, PMID: 32889259

[64] Wang L Chen G Pan Y Xia J Chen L Zhang X. China multi-ethnic cohort collaborative, association of long-term exposure to ambient air pollutants with blood lipids in Chinese adults: the China multi-ethnic cohort study. Environ Res. (2021) 197:111174. doi: 10.1016/j.envres.2021.111174, PMID: 33894235

[65] Yang BY Bloom MS Markevych I Qian ZM Vaughn MG Cummings-Vaughn LA. Exposure to ambient air pollution and blood lipids in adults: the 33 communities Chinese health study. Environ Int. (2018) 119:485–92. doi: 10.1016/j.envint.2018.07.016, PMID: 30048882

[66] Lodovici M Bigagli E. Oxidative stress and air pollution exposure. J Toxicol. (2011) 2011:487074. doi: 10.1155/2011/487074 21860622 PMC3155788

[67] Shanley RP Hayes RB Cromar KR Ito K Gordon T Ahn J. Particulate air pollution and clinical cardiovascular disease risk factors. Epidemiology. (2016) 27:291–8. doi: 10.1097/EDE.0000000000000426, PMID: 26605815 PMC4959464

[68] Chen T Jia G Wei Y Li J. Beijing ambient particle exposure accelerates atherosclerosis in ApoE knockout mice. Toxicol Lett. (2013) 223:146–53. doi: 10.1016/j.toxlet.2013.09.004, PMID: 24045146

[69] Bind MA Lepeule J Zanobetti A Gasparrini A Baccarelli A Coull BA. Air pollution and gene-specific methylation in the normative aging study: association, effect modification, and mediation analysis. Epigenetics. (2014) 9:448–58. doi: 10.4161/epi.27584, PMID: 24385016 PMC4053463

[70] Chen R Meng X Zhao A Wang C Yang C Li H. DNA hypomethylation and its mediation in the effects of fine particulate air pollution on cardiovascular biomarkers: a randomized crossover trial. Environ Int. (2016) 94:614–9. doi: 10.1016/j.envint.2016.06.026, PMID: 27397927

[71] Bevan GH Al-Kindi SG Brook R Rajagopalan S. Ambient air pollution and atherosclerosis: recent updates. Curr Atheroscler Rep. (2021) 23:63. doi: 10.1007/s11883-021-00958-9, PMID: 34417890 PMC8379601

[72] Burgess S Davies NM Thompson SG. Bias due to participant overlap in two-sample Mendelian randomization. Genet Epidemiol. (2016) 40:597–608. doi: 10.1002/gepi.21998, PMID: 27625185 PMC5082560

[73] Churg A Brauer M. Ambient atmospheric particles in the airways of human lungs. Ultrastruct Pathol. (2000) 24:353–61. PMID: 11206332 10.1080/019131200750060014

[74] Churg A Brauer M. Human lung parenchyma retains PM2.5. Am J Respir Crit Care Med. (1997) 155:2109–11. doi: 10.1164/ajrccm.155.6.9196123, PMID: 9196123

[75] Cani PD Amar J Iglesias MA Poggi M Knauf C Bastelica D. Metabolic endotoxemia initiates obesity and insulin resistance. Diabetes. (2007) 56:1761–72. doi: 10.2337/db06-1491, PMID: 17456850

[76] Liu C Ying Z Harkema J Sun Q Rajagopalan S. Epidemiological and experimental links between air pollution and type 2 diabetes. Toxicol Pathol. (2013) 41:361–73. doi: 10.1177/0192623312464531, PMID: 23104765 PMC3988529

[77] Fonceca AM Zosky GR Bozanich EM Sutanto EN Kicic A McNamara PS. Accumulation mode particles and LPS exposure induce TLR-4 dependent and independent inflammatory responses in the lung. Respir Res. (2018) 19:15. doi: 10.1186/s12931-017-0701-z, PMID: 29357863 PMC5778683

[78] Chang LT Tang CS Pan YZ Chan CC. Association of heart rate variability of the elderly with personal exposure to PM 1, PM 1-2.5, and PM 2.5-10. Bull Environ Contam Toxicol. (2007) 79:552–6. doi: 10.1007/s00128-007-9233-4, PMID: 17639313

[79] Mathew AV Yu J Guo Y Byun J Chen YE Wang L. Effect of ambient fine particulate matter air pollution and colder outdoor temperatures on high-density lipoprotein function. Am J Cardiol. (2018) 122:565–70. doi: 10.1016/j.amjcard.2018.04.061, PMID: 30005891 PMC6133768

[80] Ossoli A Favero C Vigna L Pesatori AC Bollati V Gomaraschi M. Body mass index modulates the impact of short-term exposure to air particulate matter on high-density lipoprotein function. Antioxidants (Basel). (2022) 11:1938. doi: 10.3390/antiox11101938 36290661 PMC9598765

