# Supplementary material for: Causality of particulate matter on cardiovascular diseases and cardiovascular biomarkers
Source: Front Public Health. 2023 Sep 1;11:1201479. doi: 10.3389/fpubh.2023.1201479 (PMC10507646; doi:10.3389/fpubh.2023.1201479)
Supplement: Supplementary file 1 [file Table_1.pdf]

Supplementary Material

Table 1. Genome-wide significant SNPs for PM2.5

SNP	Proxy SNP	r2 for proxy	CHR	Position	OA	EA	EAF	BETA	SE	P-value	N	R^2	F statistic
rs7573056	-	-	2	58229428	C	A	0.442	-0.011	0.002	1.50E-07	423796	0.00006	27
rs6749467	-	-	2	343517	A	G	0.466	-0.012	0.002	1.40E-08	423796	0.00008	32
rs1318845	-	-	4	153001662	C	T	0.202	-0.014	0.003	2.30E-07	423796	0.00006	27
rs72808024	-	-	5	164479164	C	A	0.149	-0.016	0.003	9.90E-08	423796	0.00007	28
rs58824859	-	-	5	137976541	C	A	0.364	0.012	0.002	1.90E-07	423796	0.00007	28
rs1372504	-	-	5	103749428	A	G	0.374	0.012	0.002	3.10E-08	423796	0.00007	30
rs12203592	-	-	6	396321	T	C	0.213	0.022	0.003	6.20E-17	423796	0.00016	67
rs77255816	-	-	6	20833602	T	C	0.037	0.031	0.006	4.20E-08	423796	0.00007	29
rs114708313	-	-	6	31329004	T	A	0.066	0.025	0.004	4.20E-08	423796	0.00007	31
rs77205736	-	-	8	10153460	T	C	0.274	0.014	0.002	2.10E-08	423796	0.00007	31
rs1537371	-	-	9	22099568	A	C	0.500	0.012	0.002	8.50E-09	423796	0.00008	32
rs11042316	-	-	11	2051631	A	G	0.258	-0.013	0.002	1.80E-07	423796	0.00007	28
rs78539764	-	-	12	12490981	C	T	0.028	-0.034	0.007	3.70E-07	423796	0.00006	26
rs11855821	-	-	15	78008843	A	G	0.283	-0.013	0.002	2.10E-07	423796	0.00006	27
rs2292156	-	-	16	49765133	T	G	0.168	0.015	0.003	3.40E-07	423796	0.00006	25
rs72642437	-	-	18	45920421	T	C	0.004	0.113	0.019	3.10E-09	423796	0.00010	42

Table 2. Genome-wide significant SNPs for PM10

SNP	Proxy SNP	r2 for proxy	CHR	Position	OA	EA	EAF	BETA	SE	P-value	N	R^2	F statistic
rs117671171	-	-	6	159246615	C	T	0.017	-0.042	0.008	4.40E-07	423796	0.00006	25
rs12203592	-	-	6	396321	C	T	0.213	0.013	0.003	4.00E-07	423796	0.00006	25
rs12192953	-	-	6	94818099	C	T	0.023	-0.039	0.007	1.60E-07	423796	0.00007	29
rs57048268	-	-	7	151623218	A	C	0.311	-0.012	0.002	1.20E-07	423796	0.00007	28
rs80230137	-	-	14	48380718	A	G	0.018	0.042	0.008	3.40E-07	423796	0.00006	26
rs4815138	-	-	20	286487	G	A	0.377	0.012	0.002	2.20E-07	423796	0.00007	28

Table 3. Instrumental variables used in MR analysis of the association between PM2.5 and AF.

SNP	Chr	Pos	EA	OA	EAF	SNP-Exposure (PM2.5)			SNP-Outcome (AF)		
						Beta	SE	p	Beta	SE	p
rs11042316	11	2051631	A	G	0.258	-0.013	0.002	1.80E-07	-0.003	0.009	0.719
rs11855821	15	78008843	A	G	0.283	-0.013	0.002	2.10E-07	-0.003	0.008	0.739
rs12203592	6	396321	T	C	0.213	0.022	0.003	6.20E-17	0.009	0.010	0.401
rs1318845	4	153001662	C	T	0.202	-0.014	0.003	2.30E-07	0.000	0.008	0.963
rs1372504	5	103749428	A	G	0.374	0.012	0.002	3.10E-08	0.000	0.007	0.969
rs2292156	16	49765133	T	G	0.168	0.015	0.003	3.40E-07	-0.011	0.009	0.221
rs58824859	5	137976541	C	A	0.364	0.012	0.002	1.90E-07	0.006	0.007	0.442
rs6749467	2	343517	A	G	0.466	-0.012	0.002	1.40E-08	-0.012	0.007	0.088
rs72642437	18	45920421	T	C	0.004	0.113	0.019	3.10E-09	-0.001	0.033	0.984

rs72808024	5	164479164	C	A	0.149	-0.016	0.003	9.90E-08	0.006	0.009	0.536
rs7573056	2	58229428	C	A	0.442	-0.011	0.002	1.50E-07	-0.002	0.007	0.732
rs77205736	8	10153460	T	C	0.274	0.014	0.002	2.10E-08	0.006	0.008	0.427
rs77255816	6	20833602	T	C	0.037	0.031	0.006	4.20E-08	0.002	0.019	0.912
rs78539764	12	12490981	C	T	0.028	-0.034	0.007	3.70E-07	0.022	0.023	0.346

Table 4. Instrumental variables used in MR analysis of the association between PM2.5 and HF.

SNP	Chr	Pos	EA	OA	EAF	SNP-Exposure (PM2.5)			SNP-Outcome (HF)		
						Beta	SE	p	Beta	SE	p
rs11042316	11	2051631	A	G	0.258	-0.013	0.002	1.80E-07	-0.011	0.010	0.237
rs114708313	6	31329004	T	A	0.066	0.025	0.004	4.20E-08	-0.030	0.016	0.058
rs11855821	15	78008843	A	G	0.283	-0.013	0.002	2.10E-07	-0.005	0.009	0.544
rs12203592	6	396321	T	C	0.213	0.022	0.003	6.20E-17	0.002	0.012	0.871
rs1318845	4	153001662	C	T	0.202	-0.014	0.003	2.30E-07	-0.002	0.010	0.844
rs1372504	5	103749428	A	G	0.374	0.012	0.002	3.10E-08	-0.002	0.008	0.804
rs2292156	16	49765133	T	G	0.168	0.015	0.003	3.40E-07	0.010	0.011	0.361
rs58824859	5	137976541	C	A	0.364	0.012	0.002	1.90E-07	-0.016	0.009	0.065
rs6749467	2	343517	A	G	0.466	-0.012	0.002	1.40E-08	-0.022	0.008	0.006
rs72642437	18	45920421	T	C	0.004	0.113	0.019	3.10E-09	0.017	0.042	0.675
rs72808024	5	164479164	C	A	0.149	-0.016	0.003	9.90E-08	0.013	0.011	0.260
rs7573056	2	58229428	C	A	0.442	-0.011	0.002	1.50E-07	-0.017	0.008	0.030
rs77205736	8	10153460	T	C	0.274	0.014	0.002	2.10E-08	-0.004	0.009	0.681
rs77255816	6	20833602	T	C	0.037	0.031	0.006	4.20E-08	0.033	0.022	0.132
rs78539764	12	12490981	C	T	0.028	-0.034	0.007	3.70E-07	-0.007	0.027	0.796

Table 5. Instrumental variables used in MR analysis of the association between PM2.5 and MI.

SNP	Chr	Pos	EA	OA	EAF	SNP-Exposure (PM2.5)			SNP-Outcome (MI)		
						Beta	SE	p	Beta	SE	p
rs11042316	11	2051631	A	G	0.258	-0.013	0.002	1.80E-07	0.002	0.013	0.900
rs114708313	6	31329004	T	A	0.066	0.025	0.004	4.20E-08	0.029	0.022	0.200
rs11855821	15	78008843	A	G	0.283	-0.013	0.002	2.10E-07	-0.023	0.012	0.057
rs1318845	4	153001662	C	T	0.202	-0.014	0.003	2.30E-07	-0.007	0.014	0.620
rs1372504	5	103749428	A	G	0.374	0.012	0.002	3.10E-08	-0.009	0.011	0.410
rs2292156	16	49765133	T	G	0.168	0.015	0.003	3.40E-07	0.026	0.014	0.065
rs58824859	5	137976541	C	A	0.364	0.012	0.002	1.90E-07	0.015	0.012	0.200
rs6749467	2	343517	A	G	0.466	-0.012	0.002	1.40E-08	-0.031	0.011	0.005
rs72642437	18	45920421	T	C	0.004	0.113	0.019	3.10E-09	0.076	0.069	0.270
rs72808024	5	164479164	C	A	0.149	-0.016	0.003	9.90E-08	-0.019	0.015	0.210
rs7573056	2	58229428	C	A	0.442	-0.011	0.002	1.50E-07	0.008	0.011	0.490
rs77205736	8	10153460	T	C	0.274	0.014	0.002	2.10E-08	0.010	0.012	0.410
rs77255816	6	20833602	T	C	0.037	0.031	0.006	4.20E-08	0.069	0.029	0.018
rs78539764	12	12490981	C	T	0.028	-0.034	0.007	3.70E-07	-0.044	0.033	0.190

Table 6. Instrumental variables used in MR analysis of the association between PM2.5 and IS.

SNP	Chr	Pos	EA	OA	EAF	SNP-Exposure (PM2.5)			SNP-Outcome (IS)		
						Beta	SE	p	Beta	SE	p
rs11042316	11	2051631	A	G	0.258	-0.013	0.002	1.80E-07	0.011	0.012	0.384
rs114708313	6	31329004	T	A	0.066	0.025	0.004	4.20E-08	-0.004	0.018	0.815
rs11855821	15	78008843	A	G	0.283	-0.013	0.002	2.10E-07	-0.009	0.011	0.422
rs12203592	6	396321	T	C	0.213	0.022	0.003	6.20E-17	0.008	0.016	0.622
rs1318845	4	153001662	C	T	0.202	-0.014	0.003	2.30E-07	0.022	0.010	0.036
rs1372504	5	103749428	A	G	0.374	0.012	0.002	3.10E-08	0.015	0.009	0.081
rs1537371	9	22099568	A	C	0.500	0.012	0.002	8.50E-09	0.049	0.008	0.000
rs2292156	16	49765133	T	G	0.168	0.015	0.003	3.40E-07	0.006	0.011	0.559
rs58824859	5	137976541	C	A	0.364	0.012	0.002	1.90E-07	0.003	0.009	0.751
rs6749467	2	343517	A	G	0.466	-0.012	0.002	1.40E-08	0.005	0.009	0.594
rs72642437	18	45920421	T	C	0.004	0.113	0.019	3.10E-09	-0.006	0.018	0.728
rs72808024	5	164479164	C	A	0.149	-0.016	0.003	9.90E-08	-0.008	0.013	0.553
rs7573056	2	58229428	C	A	0.442	-0.011	0.002	1.50E-07	0.004	0.009	0.625
rs77205736	8	10153460	T	C	0.274	0.014	0.002	2.10E-08	0.001	0.011	0.935
rs77255816	6	20833602	T	C	0.037	0.031	0.006	4.20E-08	-0.026	0.029	0.365
rs78539764	12	12490981	C	T	0.028	-0.034	0.007	3.70E-07	0.011	0.034	0.755

Table 7. Instrumental variables used in MR analysis of the association between PM2.5 and Large artery stroke.

SNP	Chr	Pos	EA	OA	EAF	SNP-Exposure (PM2.5)			SNP-Outcome (Large artery stroke)		
						Beta	SE	p	Beta	SE	p
rs11042316	11	2051631	A	G	0.258	-0.013	0.002	1.80E-07	-0.009	0.035	0.7927
rs114708313	6	31329004	T	A	0.066	0.025	0.004	4.20E-08	0.001	0.055	0.9867
rs11855821	15	78008843	A	G	0.283	-0.013	0.002	2.10E-07	-0.008	0.029	0.7864
rs12203592	6	396321	T	C	0.213	0.022	0.003	6.20E-17	-0.030	0.038	0.4354
rs1318845	4	153001662	C	T	0.202	-0.014	0.003	2.30E-07	0.033	0.033	0.3174
rs1372504	5	103749428	A	G	0.374	0.012	0.002	3.10E-08	0.031	0.025	0.2165
rs2292156	16	49765133	T	G	0.168	0.015	0.003	3.40E-07	0.043	0.036	0.2281
rs58824859	5	137976541	C	A	0.364	0.012	0.002	1.90E-07	-0.035	0.027	0.1872
rs6749467	2	343517	A	G	0.466	-0.012	0.002	1.40E-08	0.038	0.026	0.1449
rs72642437	18	45920421	T	C	0.004	0.113	0.019	3.10E-09	0.229	0.137	0.0938
rs72808024	5	164479164	C	A	0.149	-0.016	0.003	9.90E-08	0.003	0.036	0.9439
rs7573056	2	58229428	C	A	0.442	-0.011	0.002	1.50E-07	0.064	0.025	0.0103
rs77205736	8	10153460	T	C	0.274	0.014	0.002	2.10E-08	-0.018	0.028	0.5174
rs77255816	6	20833602	T	C	0.037	0.031	0.006	4.20E-08	-0.073	0.077	0.3437
rs78539764	12	12490981	C	T	0.028	-0.034	0.007	3.70E-07	0.078	0.088	0.3786

Table 8. Instrumental variables used in MR analysis of the association between PM2.5 and Small vessel stroke.

SNP	Chr	Pos	EA	OA	EAF	SNP-Exposure (PM2.5)			SNP-Outcome (Small vessel stroke)		
						Beta	SE	p	Beta	SE	p
rs11042316	11	2051631	A	G	0.258	-0.013	0.002	1.80E-07	0.026	0.033	0.426
rs114708313	6	31329004	T	A	0.066	0.025	0.004	4.20E-08	0.008	0.051	0.884
rs11855821	15	78008843	A	G	0.283	-0.013	0.002	2.10E-07	-0.028	0.027	0.297

rs12203592	6	396321	T	C	0.213	0.022	0.003	6.20E-17	-0.076	0.035	0.032
rs1318845	4	153001662	C	T	0.202	-0.014	0.003	2.30E-07	-0.005	0.030	0.857
rs1372504	5	103749428	A	G	0.374	0.012	0.002	3.10E-08	-0.001	0.024	0.983
rs1537371	9	22099568	A	C	0.500	0.012	0.002	8.50E-09	0.041	0.022	0.069
rs2292156	16	49765133	T	G	0.168	0.015	0.003	3.40E-07	-0.016	0.033	0.628
rs58824859	5	137976541	C	A	0.364	0.012	0.002	1.90E-07	0.028	0.025	0.267
rs6749467	2	343517	A	G	0.466	-0.012	0.002	1.40E-08	0.022	0.025	0.361
rs72642437	18	45920421	T	C	0.004	0.113	0.019	3.10E-09	0.166	0.154	0.281
rs72808024	5	164479164	C	A	0.149	-0.016	0.003	9.90E-08	-0.017	0.034	0.620
rs7573056	2	58229428	C	A	0.442	-0.011	0.002	1.50E-07	-0.002	0.023	0.927
rs77205736	8	10153460	T	C	0.274	0.014	0.002	2.10E-08	0.007	0.026	0.795
rs77255816	6	20833602	T	C	0.037	0.031	0.006	4.20E-08	-0.080	0.067	0.230
rs78539764	12	12490981	C	T	0.028	-0.034	0.007	3.70E-07	0.028	0.080	0.731

Table 9. Instrumental variables used in MR analysis of the association between PM2.5 and Cardioembolic stroke.

SNP	Chr	Pos	EA	OA	EAF	SNP-Exposure (PM2.5)			SNP-Outcome (Cardioembolic stroke)		
						Beta	SE	p	Beta	SE	p
rs11042316	11	2051631	A	G	0.258	-0.013	0.002	1.80E-07	0.035	0.029	0.220
rs114708313	6	31329004	T	A	0.066	0.025	0.004	4.20E-08	-0.058	0.040	0.154
rs11855821	15	78008843	A	G	0.283	-0.013	0.002	2.10E-07	0.006	0.022	0.796
rs12203592	6	396321	T	C	0.213	0.022	0.003	6.20E-17	0.007	0.032	0.815
rs1318845	4	153001662	C	T	0.202	-0.014	0.003	2.30E-07	0.009	0.025	0.708
rs1372504	5	103749428	A	G	0.374	0.012	0.002	3.10E-08	0.027	0.020	0.169
rs1537371	9	22099568	A	C	0.500	0.012	0.002	8.50E-09	0.007	0.019	0.719
rs2292156	16	49765133	T	G	0.168	0.015	0.003	3.40E-07	0.024	0.028	0.391
rs58824859	5	137976541	C	A	0.364	0.012	0.002	1.90E-07	0.023	0.021	0.254
rs6749467	2	343517	A	G	0.466	-0.012	0.002	1.40E-08	-0.061	0.020	0.003
rs72642437	18	45920421	T	C	0.004	0.113	0.019	3.10E-09	0.018	0.114	0.873
rs72808024	5	164479164	C	A	0.149	-0.016	0.003	9.90E-08	0.000	0.028	0.997
rs7573056	2	58229428	C	A	0.442	-0.011	0.002	1.50E-07	-0.003	0.020	0.897
rs77205736	8	10153460	T	C	0.274	0.014	0.002	2.10E-08	0.020	0.022	0.369
rs77255816	6	20833602	T	C	0.037	0.031	0.006	4.20E-08	-0.051	0.059	0.387
rs78539764	12	12490981	C	T	0.028	-0.034	0.007	3.70E-07	0.021	0.070	0.770

Table 10. Instrumental variables used in MR analysis of the association between PM2.5 and BMI.

SNP	Chr	Pos	EA	OA	EAF	SNP-Exposure (PM2.5)			SNP-Outcome (BMI)		
						Beta	SE	p	Beta	SE	p
rs11042316	11	2051631	A	G	0.258	-0.013	0.002	1.80E-07	0.035	0.029	0.220
rs114708313	6	31329004	T	A	0.066	0.025	0.004	4.20E-08	-0.058	0.040	0.154
rs11855821	15	78008843	A	G	0.283	-0.013	0.002	2.10E-07	0.006	0.022	0.796
rs1318845	4	153001662	C	T	0.202	-0.014	0.003	2.30E-07	0.009	0.025	0.708
rs1372504	5	103749428	A	G	0.374	0.012	0.002	3.10E-08	0.027	0.020	0.169
rs1537371	9	22099568	A	C	0.500	0.012	0.002	8.50E-09	0.007	0.019	0.719
rs2292156	16	49765133	T	G	0.168	0.015	0.003	3.40E-07	0.024	0.028	0.391

rs58824859	5	137976541	C	A	0.364	0.012	0.002	1.90E-07	0.023	0.021	0.254
rs6749467	2	343517	A	G	0.466	-0.012	0.002	1.40E-08	-0.061	0.020	0.003
rs72808024	5	164479164	C	A	0.149	-0.016	0.003	9.90E-08	0.000	0.028	0.997
rs7573056	2	58229428	C	A	0.442	-0.011	0.002	1.50E-07	-0.003	0.020	0.897
rs77205736	8	10153460	T	C	0.274	0.014	0.002	2.10E-08	0.020	0.022	0.369
rs77255816	6	20833602	T	C	0.037	0.031	0.006	4.20E-08	-0.051	0.059	0.387
rs78539764	12	12490981	C	T	0.028	-0.034	0.007	3.70E-07	0.021	0.070	0.770

Table 11. Instrumental variables used in MR analysis of the association between PM2.5 and Triglyceride.

SNP	Chr	Pos	EA	OA	EAF	SNP-Exposure (PM2.5)			SNP-Outcome (Triglyceride)		
						Beta	SE	p	Beta	SE	p
rs11855821	15	78008843	A	G	0.283	-0.013	0.002	2.10E-07	-0.007	0.006	0.181
rs12203592	6	396321	T	C	0.213	0.022	0.003	6.20E-17	0.003	0.007	0.697
rs1318845	4	153001662	C	T	0.202	-0.014	0.003	2.30E-07	0.008	0.006	0.209
rs1372504	5	103749428	A	G	0.374	0.012	0.002	3.10E-08	0.005	0.005	0.308
rs1537371	9	22099568	A	C	0.500	0.012	0.002	8.50E-09	0.004	0.005	0.402
rs2292156	16	49765133	T	G	0.168	0.015	0.003	3.40E-07	0.005	0.007	0.511
rs58824859	5	137976541	C	A	0.364	0.012	0.002	1.90E-07	0.003	0.005	0.576
rs6749467	2	343517	A	G	0.466	-0.012	0.002	1.40E-08	0.000	0.005	0.929
rs72808024	5	164482118	C	A	0.149	-0.016	0.003	9.90E-08	-0.015	0.007	0.022
rs7573056	2	58229428	C	A	0.442	-0.011	0.002	1.50E-07	-0.002	0.005	0.743
rs77205736	8	10153460	T	C	0.274	0.014	0.002	2.10E-08	0.017	0.006	0.002
rs77255816	6	20833602	T	C	0.037	0.031	0.006	4.20E-08	0.020	0.015	0.183
rs78539764	12	12490981	C	T	0.028	-0.034	0.007	3.70E-07	-0.006	0.017	0.728

Table 12. Instrumental variables used in MR analysis of the association between PM2.5 and HDL-C.

SNP	Chr	Pos	EA	OA	EAF	SNP-Exposure (PM2.5)			SNP-Outcome (HDL-C)		
						Beta	SE	p	Beta	SE	p
rs11855821	15	78008843	A	G	0.283	-0.013	0.002	2.10E-07	0.009	0.005	0.0784
rs12203592	6	396321	T	C	0.213	0.022	0.003	6.20E-17	-0.010	0.007	0.1386
rs1372504	5	103749428	A	G	0.374	0.012	0.002	3.10E-08	-0.003	0.005	0.5271
rs1537371	9	22099568	A	C	0.500	0.012	0.002	8.50E-09	-0.001	0.005	0.9000
rs2292156	16	49765133	T	G	0.168	0.015	0.003	3.40E-07	0.002	0.007	0.7697
rs58824859	5	137976541	C	A	0.364	0.012	0.002	1.90E-07	-0.002	0.005	0.6929
rs6749467	2	343517	A	G	0.466	-0.012	0.002	1.40E-08	0.000	0.005	0.9366
rs7573056	2	58229428	C	A	0.442	-0.011	0.002	1.50E-07	0.003	0.005	0.5725
rs77205736	8	10153460	T	C	0.274	0.014	0.002	2.10E-08	-0.001	0.005	0.9224
rs77255816	6	20833602	T	C	0.037	0.031	0.006	4.20E-08	-0.016	0.013	0.2323
rs78539764	12	12490981	C	T	0.028	-0.034	0.007	3.70E-07	0.013	0.016	0.4184

Table 13. Instrumental variables used in MR analysis of the association between PM2.5 and LDL-C.

SNP	Chr	Pos	EA	OA	EAF	SNP-Exposure (PM2.5)			SNP-Outcome (LDL-C)		
						Beta	SE	p	Beta	SE	p
rs11855821	15	78008843	A	G	0.283	-0.013	0.002	2.10E-07	-0.006	0.006	0.322

rs12203592	6	396321	T	C	0.213	0.022	0.003	6.20E-17	0.012	0.007	0.090
rs1318845	4	153001662	C	T	0.202	-0.014	0.003	2.30E-07	0.012	0.006	0.060
rs1372504	5	103749428	A	G	0.374	0.012	0.002	3.10E-08	0.003	0.005	0.530
rs1537371	9	22099568	A	C	0.500	0.012	0.002	8.50E-09	-0.011	0.005	0.038
rs2292156	16	49765133	T	G	0.168	0.015	0.003	3.40E-07	0.005	0.007	0.498
rs58824859	5	137976541	C	A	0.364	0.012	0.002	1.90E-07	-0.011	0.005	0.040
rs6749467	2	343517	A	G	0.466	-0.012	0.002	1.40E-08	0.002	0.005	0.640
rs72808024	5	164482118	C	A	0.149	-0.016	0.003	9.90E-08	-0.010	0.007	0.139
rs7573056	2	58229428	C	A	0.442	-0.011	0.002	1.50E-07	0.005	0.005	0.297
rs77205736	8	10153460	T	C	0.274	0.014	0.002	2.10E-08	-0.008	0.006	0.156
rs77255816	6	20833602	T	C	0.037	0.031	0.006	4.20E-08	0.021	0.016	0.170
rs78539764	12	12490981	C	T	0.028	-0.034	0.007	3.70E-07	0.017	0.017	0.320

Table 14. Instrumental variables used in MR analysis of the association between PM2.5 and Fasting insulin.

SNP	Chr	Pos	EA	OA	EAF	SNP-Exposure (PM2.5)			SNP-Outcome (Fasting insulin)		
						Beta	SE	p	Beta	SE	p
rs11042316	11	2051631	A	G	0.258	-0.013	0.002	1.80E-07	-0.002	0.003	0.418
rs114708313	6	31329004	T	A	0.066	0.025	0.004	4.20E-08	0.002	0.004	0.974
rs11855821	15	78008843	A	G	0.283	-0.013	0.002	2.10E-07	0.001	0.003	0.798
rs12203592	6	396321	T	C	0.213	0.022	0.003	6.20E-17	0.000	0.004	0.959
rs1318845	4	153001662	C	T	0.202	-0.014	0.003	2.30E-07	0.004	0.003	0.140
rs1372504	5	103749428	A	G	0.374	0.012	0.002	3.10E-08	0.003	0.002	0.411
rs1537371	9	22099568	A	C	0.500	0.012	0.002	8.50E-09	-0.004	0.002	0.008
rs2292156	16	49765133	T	G	0.168	0.015	0.003	3.40E-07	-0.008	0.003	0.003
rs58824859	5	137976541	C	A	0.364	0.012	0.002	1.90E-07	0.001	0.002	0.676
rs6749467	2	343517	A	G	0.466	-0.012	0.002	1.40E-08	-0.003	0.002	0.263
rs72642437	18	45920421	T	C	0.004	0.113	0.019	3.10E-09	0.000	0.006	0.612
rs72808024	5	164479164	C	A	0.149	-0.016	0.003	9.90E-08	0.001	0.003	0.793
rs7573056	2	58229428	C	A	0.442	-0.011	0.002	1.50E-07	0.001	0.002	0.833
rs77205736	8	10153460	T	C	0.274	0.014	0.002	2.10E-08	0.007	0.002	0.005
rs77255816	6	20833602	T	C	0.037	0.031	0.006	4.20E-08	0.003	0.006	0.815
rs78539764	12	12490981	C	T	0.028	-0.034	0.007	3.70E-07	0.001	0.008	0.916

Table 15. Instrumental variables used in MR analysis of the association between PM2.5 and Fasting glucose.

SNP	Chr	Pos	EA	OA	EAF	SNP-Exposure (PM2.5)			SNP-Outcome (Fasting glucose)		
						Beta	SE	p	Beta	SE	p
rs11042316	11	2051631	A	G	0.258	-0.013	0.002	1.80E-07	-0.002	0.003	0.355
rs114708313	6	31329004	T	A	0.066	0.025	0.004	4.20E-08	0.005	0.004	0.248
rs11855821	15	78008843	A	G	0.283	-0.013	0.002	2.10E-07	-0.001	0.002	0.707
rs12203592	6	396321	T	C	0.213	0.022	0.003	6.20E-17	-0.001	0.004	0.844
rs1318845	4	153001662	C	T	0.202	-0.014	0.003	2.30E-07	0.003	0.002	0.123
rs1372504	5	103749428	A	G	0.374	0.012	0.002	3.10E-08	-0.001	0.002	0.650
rs1537371	9	22099568	A	C	0.500	0.012	0.002	8.50E-09	0.001	0.002	0.952
rs2292156	16	49765133	T	G	0.168	0.015	0.003	3.40E-07	0.000	0.003	0.844

rs58824859	5	137976541	C	A	0.364	0.012	0.002	1.90E-07	0.005	0.002	0.022
rs72642437	18	45920421	T	C	0.004	0.113	0.019	3.10E-09	-0.003	0.006	0.849
rs72808024	5	164479164	C	A	0.149	-0.016	0.003	9.90E-08	0.001	0.003	0.588
rs7573056	2	58229428	C	A	0.442	-0.011	0.002	1.50E-07	0.001	0.002	0.565
rs77205736	8	10153460	T	C	0.274	0.014	0.002	2.10E-08	-0.002	0.002	0.767
rs77255816	6	20833602	T	C	0.037	0.031	0.006	4.20E-08	-0.001	0.005	0.563
rs78539764	12	12490981	C	T	0.028	-0.034	0.007	3.70E-07	0.007	0.006	0.154

Table 16. Instrumental variables used in MR analysis of the association between PM2.5 and Diastolic blood pressure.

SNP	Chr	Pos	EA	OA	EAF	SNP-Exposure (PM2.5)			SNP-Outcome (Diastolic blood pressure)		
						Beta	SE	p	Beta	SE	p
rs11042316	11	2051631	A	G	0.258	-0.013	0.002	1.80E-07	0.007	0.008	0.410
rs114708313	6	31329004	T	A	0.066	0.025	0.004	4.20E-08	-0.019	0.014	0.180
rs11855821	15	78008843	A	G	0.283	-0.013	0.002	2.10E-07	-0.001	0.008	0.890
rs12203592	6	396321	T	C	0.213	0.022	0.003	6.20E-17	0.002	0.008	0.840
rs1318845	4	153001662	C	T	0.202	-0.014	0.003	2.30E-07	-0.007	0.009	0.440
rs1372504	5	103749428	A	G	0.374	0.012	0.002	3.10E-08	0.008	0.007	0.260
rs1537371	9	22099568	A	C	0.500	0.012	0.002	8.50E-09	-0.009	0.007	0.180
rs2292156	16	49765133	T	G	0.168	0.015	0.003	3.40E-07	-0.006	0.009	0.530
rs58824859	5	137976541	C	A	0.364	0.012	0.002	1.90E-07	-0.007	0.007	0.320
rs6749467	2	343517	A	G	0.466	-0.012	0.002	1.40E-08	-0.011	0.007	0.130
rs72808024	5	164479164	C	A	0.149	-0.016	0.003	9.90E-08	0.008	0.010	0.440
rs7573056	2	58229428	C	A	0.442	-0.011	0.002	1.50E-07	-0.007	0.007	0.340
rs77205736	8	10153460	T	C	0.274	0.014	0.002	2.10E-08	-0.015	0.008	0.061
rs77255816	6	20833602	T	C	0.037	0.031	0.006	4.20E-08	-0.001	0.018	0.970
rs78539764	12	12490981	C	T	0.028	-0.034	0.007	3.70E-07	0.009	0.021	0.660

Table 17. Instrumental variables used in MR analysis of the association between PM2.5 and Systolic blood pressure.

SNP	Chr	Pos	EA	OA	EAF	SNP-Exposure (PM2.5)			SNP-Outcome (Systolic blood pressure)		
						Beta	SE	p	Beta	SE	p
rs11042316	11	2051631	A	G	0.258	-0.013	0.002	1.80E-07	-0.009	0.008	0.260
rs114708313	6	31329004	T	A	0.066	0.025	0.004	4.20E-08	-0.014	0.014	0.320
rs11855821	15	78008843	A	G	0.283	-0.013	0.002	2.10E-07	0.003	0.008	0.660
rs12203592	6	396321	T	C	0.213	0.022	0.003	6.20E-17	0.002	0.008	0.840
rs1318845	4	153001662	C	T	0.202	-0.014	0.003	2.30E-07	0.007	0.009	0.410
rs1372504	5	103749428	A	G	0.374	0.012	0.002	3.10E-08	0.004	0.007	0.620
rs1537371	9	22099568	A	C	0.500	0.012	0.002	8.50E-09	0.006	0.007	0.370
rs2292156	16	49765133	T	G	0.168	0.015	0.003	3.40E-07	0.000	0.009	0.960
rs58824859	5	137976541	C	A	0.364	0.012	0.002	1.90E-07	-0.003	0.007	0.700
rs6749467	2	343517	A	G	0.466	-0.012	0.002	1.40E-08	-0.002	0.007	0.760
rs72808024	5	164479164	C	A	0.149	-0.016	0.003	9.90E-08	0.005	0.010	0.650
rs7573056	2	58229428	C	A	0.442	-0.011	0.002	1.50E-07	-0.003	0.007	0.690
rs77205736	8	10153460	T	C	0.274	0.014	0.002	2.10E-08	-0.011	0.008	0.160
rs77255816	6	20833602	T	C	0.037	0.031	0.006	4.20E-08	0.001	0.018	0.970

rs78539764	12	12490981	C	T	0.028	-0.034	0.007	3.70E-07	0.014	0.021	0.520

Table 18. Instrumental variables used in MR analysis of the association between PM10 and AF.

SNP	Chr	Pos	EA	OA	EAF	SNP-Exposure (PM10)			SNP-Outcome (AF)		
						Beta	SE	p	Beta	SE	p
rs117671171	6	159246615	T	C	0.017	-0.042	0.008	4.40E-07	0.033	0.030	0.277
rs12192953	6	94818099	T	C	0.023	-0.039	0.007	1.60E-07	-0.074	0.025	0.003
rs12203592	6	396321	T	C	0.213	0.013	0.003	4.00E-07	0.009	0.010	0.401
rs4815138	20	286487	A	G	0.377	0.012	0.002	2.20E-07	0.013	0.007	0.059
rs57048268	7	151623218	C	A	0.311	-0.012	0.002	1.20E-07	0.005	0.008	0.543
rs80230137	14	48380718	G	A	0.018	0.042	0.008	3.40E-07	-0.004	0.025	0.870

Table 19. Instrumental variables used in MR analysis of the association between PM10 and HF.

SNP	Chr	Pos	EA	OA	EAF	SNP-Exposure (PM10)			SNP-Outcome (HF)		
						Beta	SE	p	Beta	SE	p
rs117671171	6	159246615	T	C	0.017	-0.042	0.008	4.40E-07	0.002	0.033	0.942
rs12192953	6	94818099	T	C	0.023	-0.039	0.007	1.60E-07	-0.072	0.029	0.013
rs12203592	6	396321	T	C	0.213	0.013	0.003	4.00E-07	0.002	0.012	0.871
rs4815138	20	286487	A	G	0.377	0.012	0.002	2.20E-07	0.008	0.008	0.334
rs57048268	7	151623218	C	A	0.311	-0.012	0.002	1.20E-07	-0.005	0.009	0.586
rs80230137	14	48380718	G	A	0.018	0.042	0.008	3.40E-07	0.041	0.034	0.229

Table 20. Instrumental variables used in MR analysis of the association between PM10 and MI.

SNP	Chr	Pos	EA	OA	EAF	SNP-Exposure (PM10)			SNP-Outcome (MI)		
						Beta	SE	p	Beta	SE	p
rs117671171	6	159246615	T	C	0.017	-0.042	0.008	4.40E-07	0.000	0.042	0.990
rs12192953	6	94818099	T	C	0.023	-0.039	0.007	1.60E-07	0.035	0.037	0.350
rs12203592	6	396321	T	C	0.213	0.013	0.003	4.00E-07	-0.011	0.014	0.420
rs4815138	20	286487	A	G	0.377	0.012	0.002	2.20E-07	0.003	0.011	0.770
rs57048268	7	151623218	C	A	0.311	-0.012	0.002	1.20E-07	-0.011	0.012	0.330
rs80230137	14	48380718	G	A	0.018	0.042	0.008	3.40E-07	0.015	0.038	0.690

Table 21. Instrumental variables used in MR analysis of the association between PM10 and IS.

SNP	Chr	Pos	EA	OA	EAF	SNP-Exposure (PM10)			SNP-Outcome (IS)		
						Beta	SE	p	Beta	SE	p
rs12192953	6	94818099	T	C	0.023	-0.039	0.007	1.60E-07	-0.080	0.039	0.043
rs12203592	6	396321	T	C	0.213	0.013	0.003	4.00E-07	0.008	0.016	0.622
rs4815138	20	286487	A	G	0.377	0.012	0.002	2.20E-07	-0.004	0.009	0.676
rs57048268	7	151623218	C	A	0.311	-0.012	0.002	1.20E-07	-0.006	0.011	0.595
rs80230137	14	48380718	G	A	0.018	0.042	0.008	3.40E-07	0.057	0.026	0.030

Table 22. Instrumental variables used in MR analysis of the association between PM10 and Large artery stroke.

SNP	Chr	Pos	EA	OA	EAF	SNP-Exposure (PM10)			SNP-Outcome (Large artery stroke)		
						Beta	SE	p	Beta	SE	p
rs117671171	6	159246615	T	C	0.017	-0.042	0.008	4.40E-07	0.274	0.107	0.010
rs12192953	6	94818099	T	C	0.023	-0.039	0.007	1.60E-07	-0.027	0.102	0.790
rs12203592	6	396321	T	C	0.213	0.013	0.003	4.00E-07	-0.030	0.038	0.435
rs4815138	20	286487	A	G	0.377	0.012	0.002	2.20E-07	-0.033	0.027	0.232
rs57048268	7	151623218	C	A	0.311	-0.012	0.002	1.20E-07	-0.024	0.033	0.467
rs80230137	14	48380718	G	A	0.018	0.042	0.008	3.40E-07	-0.036	0.104	0.732

Table 23. Instrumental variables used in MR analysis of the association between PM10 and Small vessel stroke.

SNP	Chr	Pos	EA	OA	EAF	SNP-Exposure (PM10)			SNP-Outcome (Small vessel stroke)		
						Beta	SE	p	Beta	SE	p
rs117671171	6	159246615	T	C	0.017	-0.042	0.008	4.40E-07	-0.090	0.108	0.405
rs12192953	6	94818099	T	C	0.023	-0.039	0.007	1.60E-07	0.070	0.089	0.431
rs12203592	6	396321	T	C	0.213	0.013	0.003	4.00E-07	-0.076	0.035	0.032
rs4815138	20	286487	A	G	0.377	0.012	0.002	2.20E-07	0.033	0.025	0.189
rs57048268	7	151623218	C	A	0.311	-0.012	0.002	1.20E-07	0.034	0.030	0.259
rs80230137	14	48380718	G	A	0.018	0.042	0.008	3.40E-07	0.014	0.096	0.880

Table 24. Instrumental variables used in MR analysis of the association between PM10 and Cardioembolic stroke.

SNP	Chr	Pos	EA	OA	EAF	SNP-Exposure (PM10)			SNP-Outcome (Cardioembolic stroke)		
						Beta	SE	p	Beta	SE	p
rs117671171	6	159246615	T	C	0.017	-0.042	0.008	4.40E-07	0.116	0.095	0.222
rs12192953	6	94818099	T	C	0.023	-0.039	0.007	1.60E-07	-0.108	0.084	0.198
rs12203592	6	396321	T	C	0.213	0.013	0.003	4.00E-07	0.007	0.032	0.815
rs4815138	20	286487	A	G	0.377	0.012	0.002	2.20E-07	-0.028	0.021	0.175
rs57048268	7	151623218	C	A	0.311	-0.012	0.002	1.20E-07	-0.027	0.025	0.282
rs80230137	14	48380718	G	A	0.018	0.042	0.008	3.40E-07	0.012	0.089	0.895

Table 25. Instrumental variables used in MR analysis of the association between PM10 and BMI.

SNP	Chr	Pos	EA	OA	EAF	SNP-Exposure (PM10)			SNP-Outcome (BMI)		
						Beta	SE	p	Beta	SE	p
rs117671171	6	159246615	T	C	0.017	-0.042	0.008	4.40E-07	0.116	0.095	0.222
rs12192953	6	94818099	T	C	0.023	-0.039	0.007	1.60E-07	-0.108	0.084	0.198
rs12203592	6	396321	T	C	0.213	0.013	0.003	4.00E-07	0.007	0.032	0.815
rs4815138	20	286487	A	G	0.377	0.012	0.002	2.20E-07	-0.028	0.021	0.175
rs57048268	7	151623218	C	A	0.311	-0.012	0.002	1.20E-07	-0.027	0.025	0.282
rs80230137	14	48380718	G	A	0.018	0.042	0.008	3.40E-07	0.012	0.089	0.895

Table 26. Instrumental variables used in MR analysis of the association between PM10 and Triglyceride.

SNP	Chr	Pos	EA	OA	EAF	SNP-Exposure (PM10)			SNP-Outcome (Triglyceride)		
						Beta	SE	p	Beta	SE	p
rs117671171	6	159246615	T	C	0.017	-0.042	0.008	4.40E-07	-0.005	0.022	0.825
rs12192953	6	94818099	T	C	0.023	-0.039	0.007	1.60E-07	-0.002	0.017	0.910

rs12203592	6	396321	T	C	0.213	0.013	0.003	4.00E-07	0.003	0.007	0.697
rs4815138	20	286487	A	G	0.377	0.012	0.002	2.20E-07	0.004	0.005	0.442
rs57048268	7	151623218	C	A	0.311	-0.012	0.002	1.20E-07	-0.007	0.006	0.226

Table 27. Instrumental variables used in MR analysis of the association between PM10 and HDL-C.

SNP	Chr	Pos	EA	OA	EAF	SNP-Exposure (PM10)			SNP-Outcome (HDL-C)		
						Beta	SE	p	Beta	SE	p
rs12192953	6	94818099	T	C	0.023	-0.039	0.007	1.60E-07	0.038	0.016	0.020
rs12203592	6	396321	T	C	0.213	0.013	0.003	4.00E-07	-0.010	0.007	0.139
rs4815138	20	286487	A	G	0.377	0.012	0.002	2.20E-07	0.001	0.005	0.913
rs57048268	7	151623218	C	A	0.311	-0.012	0.002	1.20E-07	0.008	0.005	0.120

Table 28. Instrumental variables used in MR analysis of the association between PM10 and LDL-C.

SNP	Chr	Pos	EA	OA	EAF	SNP-Exposure (PM10)			SNP-Outcome (LDL-C)		
						Beta	SE	p	Beta	SE	p
rs117671171	6	159246615	T	C	0.017	-0.042	0.008	4.40E-07	-0.028	0.022	0.202
rs12192953	6	94818099	T	C	0.023	-0.039	0.007	1.60E-07	0.004	0.017	0.814
rs12203592	6	396321	T	C	0.213	0.013	0.003	4.00E-07	0.012	0.007	0.090
rs4815138	20	286487	A	G	0.377	0.012	0.002	2.20E-07	0.007	0.005	0.162
rs57048268	7	151623218	C	A	0.311	-0.012	0.002	1.20E-07	0.004	0.006	0.528

Table 29. Instrumental variables used in MR analysis of the association between PM10 and Fasting insulin.

SNP	Chr	Pos	EA	OA	EAF	SNP-Exposure (PM10)			SNP-Outcome (Fasting insulin)		
						Beta	SE	p	Beta	SE	p
rs117671171	6	159246615	T	C	0.017	-0.042	0.008	4.40E-07	0.003	0.009	0.404
rs12192953	6	94818099	T	C	0.023	-0.039	0.007	1.60E-07	0.003	0.009	0.949
rs12203592	6	396321	T	C	0.213	0.013	0.003	4.00E-07	0.000	0.004	0.959
rs4815138	20	286487	A	G	0.377	0.012	0.002	2.20E-07	0.003	0.002	0.305
rs57048268	7	151623218	C	A	0.311	-0.012	0.002	1.20E-07	0.005	0.003	0.326
rs80230137	14	48380718	G	A	0.018	0.042	0.008	3.40E-07	0.011	0.008	0.379

Table 30. Instrumental variables used in MR analysis of the association between PM10 and Fasting glucose.

SNP	Chr	Pos	EA	OA	EAF	SNP-Exposure (PM10)			SNP-Outcome (Fasting glucose)		
						Beta	SE	p	Beta	SE	p
rs117671171	6	159246615	T	C	0.017	-0.042	0.008	4.40E-07	0.007	0.008	0.464
rs12192953	6	94818099	T	C	0.023	-0.039	0.007	1.60E-07	0.004	0.007	0.444
rs12203592	6	396321	T	C	0.213	0.013	0.003	4.00E-07	-0.001	0.004	0.844
rs4815138	20	286487	A	G	0.377	0.012	0.002	2.20E-07	0.000	0.002	0.781
rs57048268	7	151623218	C	A	0.311	-0.012	0.002	1.20E-07	0.002	0.002	0.472
rs80230137	14	48380718	G	A	0.018	0.042	0.008	3.40E-07	-0.008	0.007	0.121

Table 31. Instrumental variables used in MR analysis of the association between PM10 and Diastolic blood pressure.

SNP	Chr	Pos	EA	OA	EAF	SNP-Exposure (PM10)			SNP-Outcome (Diastolic blood pressure)		
						Beta	SE	p	Beta	SE	p

rs117671171	6	159246615	T	C	0.017	-0.042	0.008	4.40E-07	0.025	0.027	0.350
rs12192953	6	94818099	T	C	0.023	-0.039	0.007	1.60E-07	0.004	0.023	0.880
rs12203592	6	396321	T	C	0.213	0.013	0.003	4.00E-07	0.002	0.008	0.840
rs4815138	20	286487	A	G	0.377	0.012	0.002	2.20E-07	0.000	0.007	0.950
rs57048268	7	151623218	C	A	0.311	-0.012	0.002	1.20E-07	-0.006	0.008	0.410
rs80230137	14	48380718	G	A	0.018	0.042	0.008	3.40E-07	0.018	0.028	0.530

Table 32. Instrumental variables used in MR analysis of the association between PM10 and Systolic blood pressure.

SNP	Chr	Pos	EA	OA	EAF	SNP-Exposure (PM10)			SNP-Outcome (Systolic blood pressure)		
						Beta	SE	p	Beta	SE	p
rs117671171	6	159246615	T	C	0.017	-0.042	0.008	4.40E-07	0.046	0.027	0.086
rs12192953	6	94818099	T	C	0.023	-0.039	0.007	1.60E-07	0.015	0.023	0.520
rs12203592	6	396321	T	C	0.213	0.013	0.003	4.00E-07	0.002	0.008	0.840
rs4815138	20	286487	A	G	0.377	0.012	0.002	2.20E-07	0.000	0.007	0.980
rs57048268	7	151623218	C	A	0.311	-0.012	0.002	1.20E-07	0.000	0.008	0.990
rs80230137	14	48380718	G	A	0.018	0.042	0.008	3.40E-07	-0.008	0.007	0.121

A

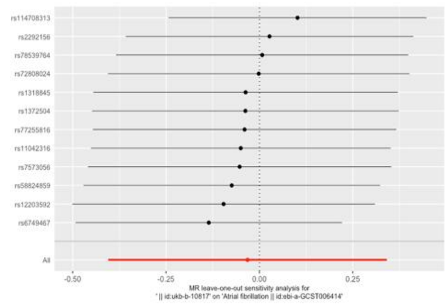

B

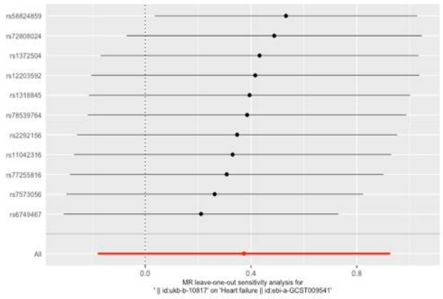

C

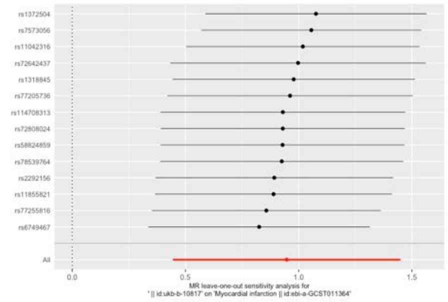

D

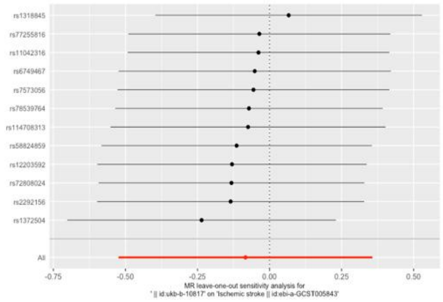

E

F

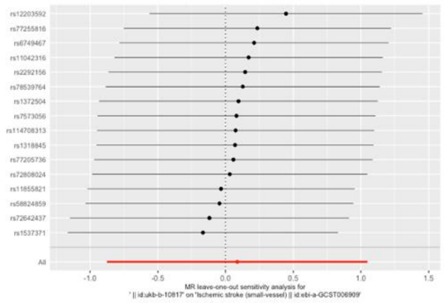

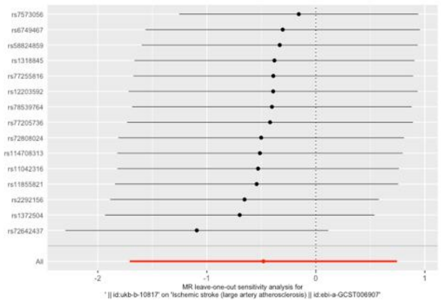

G

H

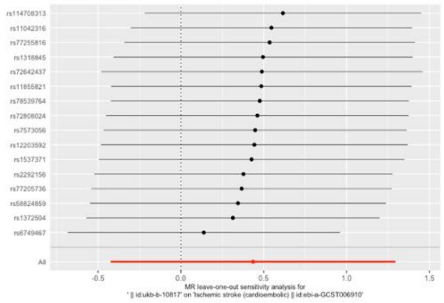

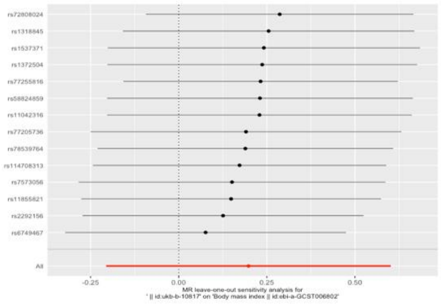

I

J

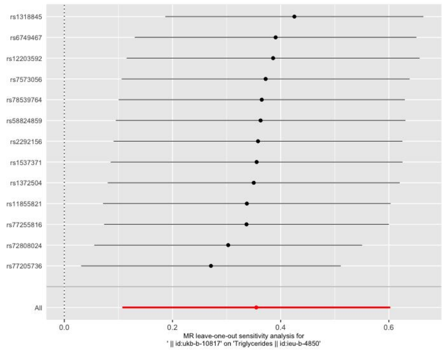

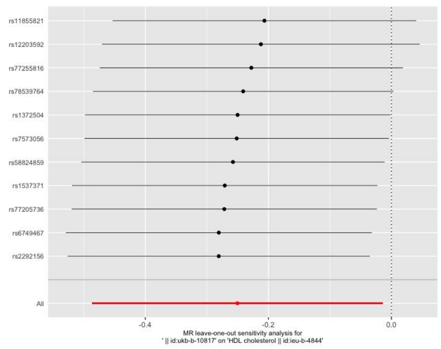

K

L

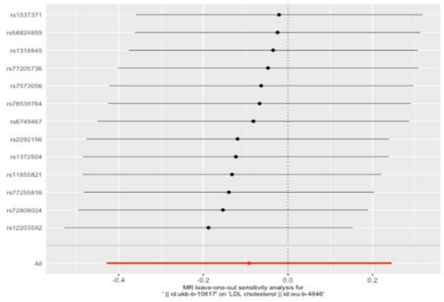

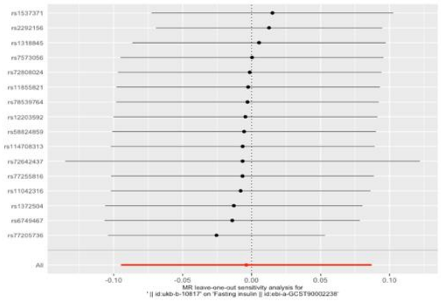

M

N

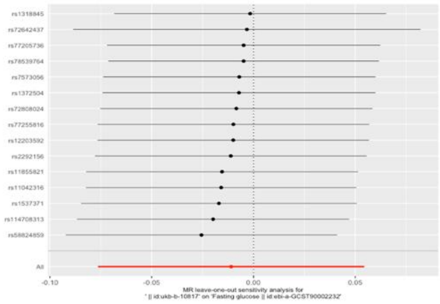

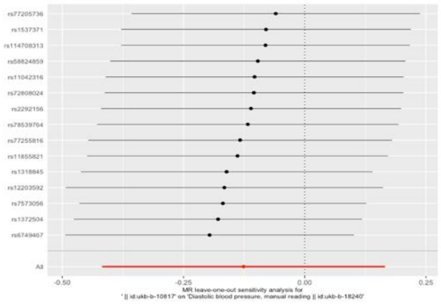

O

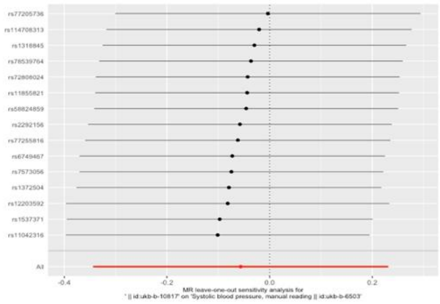

(A) Leave-one-out sensitivity analysis for the effect of PM2.5 on AF.
(B) Leave-one-out sensitivity analysis for the effect of PM2.5 on HF.
(C) Leave-one-out sensitivity analysis for the effect of PM2.5 on MI.
(D) Leave-one-out sensitivity analysis for the effect of PM2.5 on IS.
(E) Leave-one-out sensitivity analysis for the effect of PM2.5 on Small vessel stroke.
(F) Leave-one-out sensitivity analysis for the effect of PM2.5 on Large artery stroke.
(G) Leave-one-out sensitivity analysis for the effect of PM2.5 on Cardioembolic stroke.
(H) Leave-one-out sensitivity analysis for the effect of PM2.5 on BMI.
(I) Leave-one-out sensitivity analysis for the effect of PM2.5 on TG.
(J) Leave-one-out sensitivity analysis for the effect of PM2.5 on HDL-C.
(K) Leave-one-out sensitivity analysis for the effect of PM2.5 on LDL-C.
(L) Leave-one-out sensitivity analysis for the effect of PM2.5 on Fasting insulin.
(M) Leave-one-out sensitivity analysis for the effect of PM2.5 on Fasting glucose.
(N) Leave-one-out sensitivity analysis for the effect of PM2.5 on DBP.
(O) Leave-one-out sensitivity analysis for the effect of PM2.5 on SBP.

A

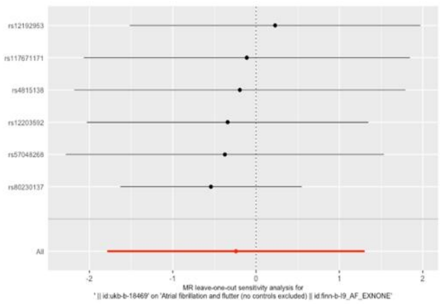

B

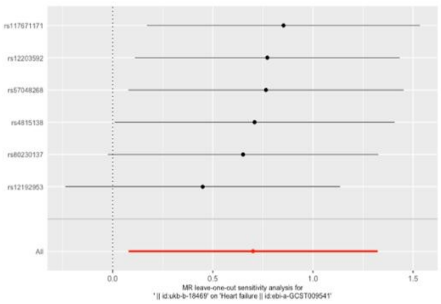

C

D

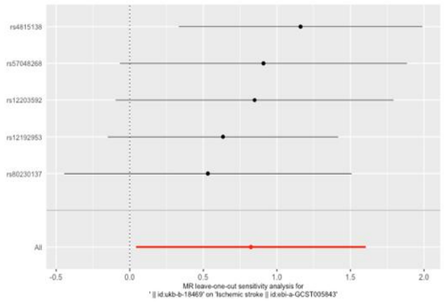
rs4815138
rs57048268
rs12203592
rs12192953
rs80230137
All
-0.5
0.0
0.5
1.0
1.5
2.0
MR leave-one-out sensitivity analysis for
' || id:ukb-b-18469' on 'Ischemic stroke || id:ebi-a-GCST005843'

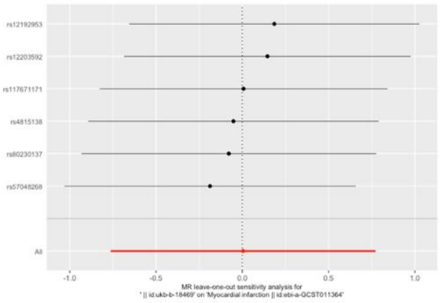
rs12192953
rs12203592
rs117671171
rs4815138
rs80230137
rs57048268
All
-1.0
-0.5
0.0
0.5
1.0
MR leave-one-out sensitivity analysis for
' || id:ukb-b-18469' on 'Myocardial infarction || id:ebi-a-GCST011364'

E

F

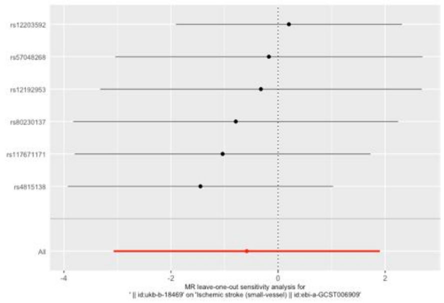
rs12203592
rs57048268
rs12192953
rs80230137
rs117671171
rs4815138
All
-4
-2
0
2
MR leave-one-out sensitivity analysis for
' || id:ukb-b-18469' on 'Ischemic stroke (small-vessel) || id:ebi-a-GCST006909'

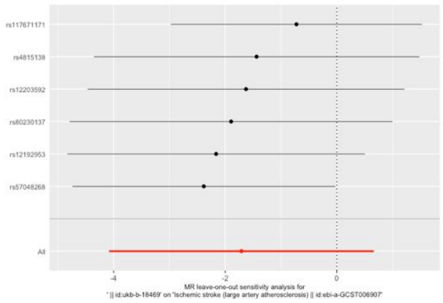
rs117671171
rs4815138
rs12203592
rs80230137
rs12192953
rs57048268
All
-4
-2
0
MR leave-one-out sensitivity analysis for
' || id:ukb-b-18469' on 'Ischemic stroke (large artery atherosclerosis) || id:ebi-a-GCST006907'

G

H

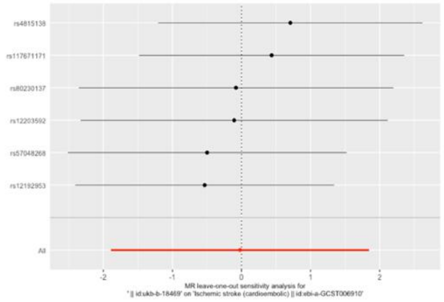
rs4815138
rs117671171
rs80230137
rs12203592
rs57048268
rs12192953
All
-2
-1
0
1
2
MR leave-one-out sensitivity analysis for
' || id:ukb-b-18469' on 'Ischemic stroke (cardioembolic) || id:ebi-a-GCST006910'

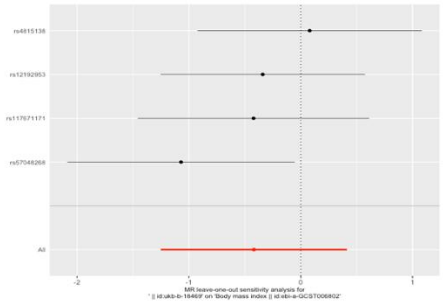
rs4815138
rs12192953
rs117671171
rs57048268
All
-2
-1
0
1
MR leave-one-out sensitivity analysis for
' || id:ukb-b-18469' on 'Body mass index || id:ebi-a-GCST006802'

I

J

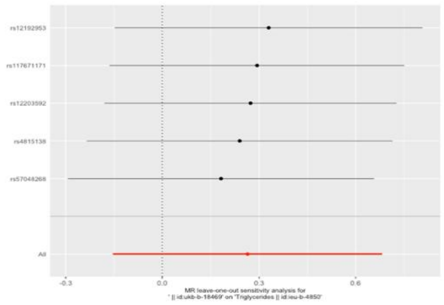
rs12192953
rs117671171
rs12203592
rs4815138
rs57048268
All
-0.3
0.0
0.3
0.6
MR leave-one-out sensitivity analysis for
' || id:ukb-b-18469' on 'Triglycerides || id:ieu-b-4850'

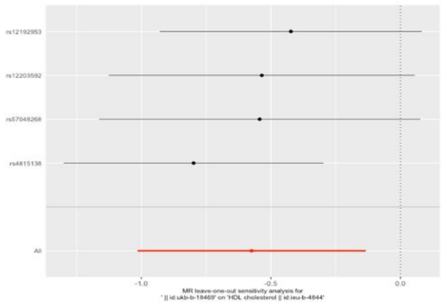
rs12192953
rs12203592
rs57048268
rs4815138
All
-1.0
-0.5
0.0
MR leave-one-out sensitivity analysis for
' || id:ukb-b-18469' on 'HDL cholesterol || id:ieu-b-4844'

K

L

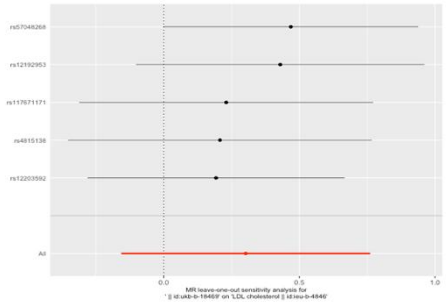

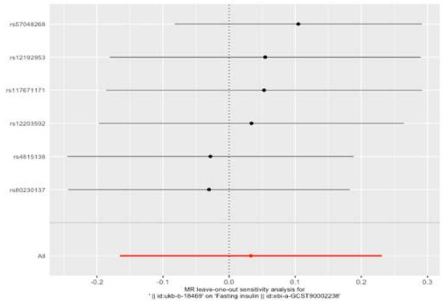

M

N

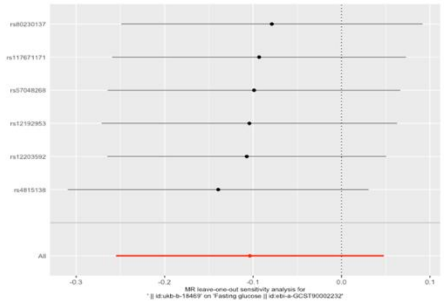

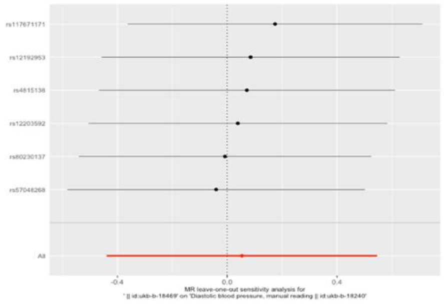

O

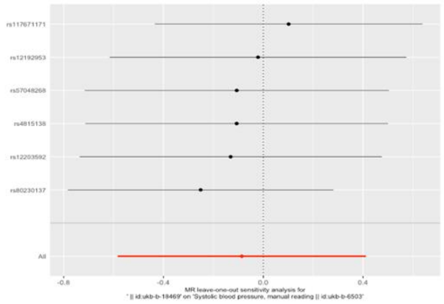

(A) Leave-one-out sensitivity analysis for the effect of PM10 on AF.

(B) Leave-one-out sensitivity analysis for the effect of PM10 on HF.

(C) Leave-one-out sensitivity analysis for the effect of PM10 on IS.

(D) Leave-one-out sensitivity analysis for the effect of PM10 on MI.

(E)Leave-one-out sensitivity analysis for the effect of PM10 on Small vessel stroke.

(F)Leave-one-out sensitivity analysis for the effect of PM10 on Large artery stroke.

(G)Leave-one-out sensitivity analysis for the effect of PM10 on Cardioembolic stroke.

(H)Leave-one-out sensitivity analysis for the effect of PM2.5 on BMI.

(I) Leave-one-out sensitivity analysis for the effect of PM2.5 on TG.

(J) Leave-one-out sensitivity analysis for the effect of PM2.5 on HDL-C.

(K) Leave-one-out sensitivity analysis for the effect of PM2.5 on LDL-C.

(L) Leave-one-out sensitivity analysis for the effect of PM2.5 on Fasting insulin.

(M)Leave-one-out sensitivity analysis for the effect of PM2.5 on Fasting glucose.

(N) Leave-one-out sensitivity analysis for the effect of PM2.5 on DBP.

(O) Leave-one-out sensitivity analysis for the effect of PM2.5 on SBP.

Table 33. Mendelian randomization estimates between PM2.5 and cardiovascular biomarkers.

outcomes	IVW (random effects)		weighted median		MR-egger	
	OR (95CI)	P value	OR (95CI)	P value	OR (95CI)	P value
BMI	1.219 (0.814-1.825)	0.337	0.989 (0.620-1.825)	0.962	0.456 (0.026-7.880)	0.599
TG	1.426 (1.113-1.827)	0.005	1.327 (0.969-1.817)	0.077	1.528 (0.551-4.237)	0.433
HDL-C	0.779 (0.615-0.986)	0.038	0.788 (0.585-1.061)	0.116	0.534 (0.230-1.242)	0.179
LDL-C	0.913 (0.651-1.278)	0.594	0.970 (0.676-1.391)	0.867	2.588 (0.764-8.763)	0.155
Fasting insulin	0.996 (0.910-1.091)	0.933	1.000 (0.910-1.099)	0.996	1.002 (0.862-1.165)	0.982
Fasting glucose	0.989 (0.926-1.056)	0.742	0.976 (0.887-1.073)	0.611	0.967 (0.867-1.080)	0.564
DBP	0.881 (0.658-1.180)	0.397	0.965 (0.643-1.449)	0.864	0.661 (0.230-1.897)	0.864
SBP	0.945 (0.709-1.260)	0.701	1.027 (0.700-1.506)	0.892	0.654 (0.237-1.803)	0.427

Table 34. Mendelian randomization estimates between PM10 and cardiovascular biomarkers.

outcomes	IVW (random effects)		weighted median		MR-egger	
	OR (95CI)	P value	OR (95CI)	P value	OR (95CI)	P value
BMI	0.658 (0.286-1.512)	0.324	0.729 (0.266-2.002)	0.540	0.283 (0.002-44.622)	0.673
TG	1.302 (0.857-1.978)	0.216	1.252 (0.732-2.143)	0.412	0.939 (0.351-2.510)	0.908
HDL-C	0.563 (0.362-0.874)	0.011	0.502 (0.289-0.872)	0.015	0.270 (0.080-0.908)	0.169
LDL-C	1.353 (0.855-2.140)	0.197	1.407 (0.840-2.359)	0.195	1.177 (0.344-4.026)	0.812
Fasting insulin	1.033 (0.848-1.260)	0.745	1.007 (0.805-1.261)	0.950	1.074 (0.689-1.674)	0.769
Fasting glucose	0.902 (0.775-1.049)	0.180	0.887 (0.736-1.068)	0.204	0.813 (0.599-1.103)	0.254
DBP	1.055 (0.645-1.727)	0.831	1.037 (0.568-1.894)	0.905	0.801 (0.276-2.320)	0.703
SBP	0.918 (0.558-1.509)	0.735	1.014 (0.550-1.871)	0.965	0.708 (0.221-2.273)	0.593
